# Genomic Intelligence as Über Bio-Cybersecurity: The Gödel Sentence in Immuno-Cognitive Systems

**DOI:** 10.3390/e23040405

**Published:** 2021-03-29

**Authors:** Sheri M. Markose

**Affiliations:** Department of Economics, University of Essex, Essex C04 3SQ, UK; scher@essex.ac.uk

**Keywords:** genomic intelligence, biology as computation, mirror systems of Self-Ref and Self-Rep, liar/hacker/antigen, offline simulation, immuno-cognitive system, strategic innovation, novelty

## Abstract

This paper gives formal foundations and evidence from gene science in the post Barbara McClintock era that the Gödel Sentence, far from being an esoteric construction in mathematical logic, is ubiquitous in genomic intelligence that evolved with multi-cellular life. Conditions uniquely found in the Adaptive Immune System (AIS) and Mirror Neuron System (MNS), termed the genomic immuno-cognitive system, coincide with three building blocks in computation theory of Gödel, Turing and Post (G-T-P). (i) Biotic elements have unique digital identifiers with gene codes executing 3D self-assembly for morphology and regulation of the organism using the recursive operation of Self-Ref (Self-Reference) with the *other* being a self-referential projection of self. (ii) A parallel offline simulation meta/mirror environment in 1–1 relation to online machine executions of self-codes gives G-T-P Self-Rep (Self-Representation). (iii) This permits a digital biotic entity to self-report that it is under attack by a biotic malware or non-self antigen in the format of the Gödel sentence, resulting in the “smarts” for contextual novelty production. The proposed unitary G-T-P recursive machinery in AIS and in MNS for social cognition yields a new explanation that the Interferon Gamma factor, known for friend-foe identification in AIS, is also integral to social behaviors. New G-T-P bio-informatics of AIS and novel anti-body production is given with interesting testable implications for COVID-19 pathology.

## 1. Introduction

Increasingly, since the epochal discovery of the digital code of the genome that uses a near universal 4 letter base (A, C, G, T/U), there has been a call to arms for a unified computational model for biology [1]. There is a push for models for biotic operations on encoded information as digital computation in a literal sense and not just as metaphors [2,3,4,5]. Nurse [6] has been influential in suggesting that there is a need for a logic tool kit that represents natural genomic digital information processing, referred to as genomic intelligence, and for a framework that provides links from this to the cellular biochemistry [7]. Following the pioneering work of Ramakrishnan [8], there are now detailed models of bio-molecular computing evidenced in ribosomal RNA that take after the ideal model of computing of Turing Machines [9,10]. The ribosomal machine execution of encoded procedures with its extreme commitment to fidelity and quality control with inbuilt proof reading and repair mechanisms in place [11,12], has been referred to as Read only Memory (ROM) digital processing [13] as it does not permit endogenous change.

It is widely acknowledged that Barbara McClintock [14] with her Noble Prize-winning work began to dislodge the view that genomic novelty is not just the result of random mutations in replication or transcription errors. McClintock’s epochal discovery of transposons and retrotransposons in the genome is, respectively, associated with two basic computational operations (see [15,16,17]) of “copy and paste/print” and “cut and paste” of digital information. McClintock [14] pioneered the notion of the “dynamic genome” [18] that can sense, respond and adapt to stressful conditions for the genome. These capabilities of sensing and even cognition at the level of biotic elements are purported to creatively respond with exaptation of already extant functional gene codes, using software from transposable elements, to produce viable and novel solutions under conditions of stress.

The ancient ancestry of transposable elements has been traced to viral software from the RNA virus or DNA virus [19]. Indeed, in his review of [20], Dyson [21] uses an apt and colorful description of the Faustian pact involved in life itself in that the replicative code in the DNA was the result “of a digital parasite incorporated into the analog metabolism of its original host”. This is a view held by others [22,23]. Thus, transposable elements, aka jumping genes, have been found to give endogenous flexibility to genetic material and is at the heart of eukaryote evolvability, Fedoroff [24] (see [25]). While this has set in motion what Shapiro [13,26] has called the read-write enhancements to the core ROM only components of the genome, the viral software has to be kept under tight check due to its potential malign activity [27,28].

At the frontiers of molecular biology, bio-molecular computing [29,30], bio-informatics, advances in bio-inspired AI [31,32] and with research groups organized around biology as computation [33], the search is on to fill the lacunae in information processing in complex genomic digital systems. The focus of this paper is on genomic intelligence found in eukaryote evolution that facilitates complex interactions between self and other, along with the endogenous evolvability capable of generating context-dependent adaptive and novel phenotypic variation while maintaining genomic stability in the face of biotic viruses and pathogens. The considerable data deluge inherent to the sheer complexity of the biological evidence that has followed the great strides made in gene science and neuroscience, has caused Brenner [3] to point out the lack of a unifying theoretical framework (see [34]).

The objective of this paper is to show that a unifying framework for genomic intelligence which involves biotic digital information processing in both the immune and neural systems will require the full gamut of foundational work on digital computation or the recursive function theory of Gödel [35], Turing [36], and Post [37] (G-T-P hereafter). The discourse has to go beyond a model of Turing machine execution of a gene code and also envisage the organization of encoded information within the framework of a G-T-P formal system wedded to the principle of consistency [38], and capable of identifying the negation operation or malware alterations of tissue specific gene codes. For this, as first formalized in Markose’s Keynote talk [39] and backed up by evidence from gene and neurosciences also reported here, the genomic immuno-cognitive system acquired the wherewithal to encode the Gödel sentence so that a biotic code can self-report that it is under attack and the precise code of the hacker/antigen can be identified, along with the production of novel responses. For this, (1) the *first* condition in the Gödelization of the genomic system is the necessity of unique digital identifiers of encoded information a.k.a. Gödel numbers (g.ns). An important set of recursive operations on them involves 3D self-assembly for the morphology and regulation of the organism. The notion of a program, *g*, that builds the machine that is then instructed to implement/run the same code *g*, takes the form of self-assembly operation called Self-Ref (Self Reference) which typically uses the notation ***Diag***(.) in recursive function theory. (2) The *second* necessary condition for a digital system to encode the Gödel sentence is a mirror system that yields a 1–1 mapping between online machine executions and a meta/offline recording system on which offline simulation can be done. For this, the Rogers meta-representation theorem [16] will be used. This has been called Self-Rep (Self-Representation, see [40,41]), which also permits a self-referential alignment between the self and other using the Gödel substitution function. (3) The *third* G-T-P condition involves the identification of the Liar qua malware/hacker which requires the wherewithal of the recursive machinery involving the Second Recursion Theorem [42,43] for the fixed point of the former to take the form of the Gödel sentence at the level of the biotic element targeted by the malware. Without this extensive G-T-P recursive machinery being hardwired into the code-based genomic system, the latter will be vulnerable to biotic malware, incapable of novelty production, and thereby being entrained within an inflexible repertoire.

Except for Markose [44,45] and Tsuda [46], there has not been an explicit acknowledgement of the necessity of a precise Gödel meta-mirror system that is parallel to codes from online machine executions by self in a model of cognition. The epochal discovery of the Mirror Neuron System (MNS) in the brain by the Parma Group [47,48,49] in the 1990s led to the characterization of MNS in the facilitation of social cognition of other’s actions by self-referential reuse of codes from the neuronal firings from the agent’s own motor and sensory activity (see [50,51]). The latter have been called canonical neurons [52]. Tsuda [46] has stated how neural systems which need to process a self-referential description use the recursive mappings of a mirror system as in the mathematics of the Gödel’s incompleteness theorem: “When neural systems process a self-referential description, they may first have *to make a copy* (italics added) of the object of self-reference and then refer to this copy. This two-stage formulation can be realized mathematically in the proof of Gödel’s incompleteness theorem through the processes of projecting mathematical statements to natural numbers and of referring to meta-mathematical statements by providing mathematical statements about such numbers. The presence of mirror neurons in animal brains or mirror neuron systems in human brains may also be a realization of the above two-stage formulation in brains, because mirror neurons, or mirror-neuron systems, can be activated, not only by behavior in others similar to one’s own behavior, but also by one’s own behavior.”

However, [46] did not extend the necessity of the Gödel framework for the identification of hostile viral software. This has been variously referred to as “Viruses in Turing’s Garden” (see [42,43,53]), the Liar strategy [44,45] and by [54] on the necessity of self-referential structures involving inverter/negator machines for the generation of the undecidable syntactic objects associated with recursive novelty production. Markose [45] cites the significance of the experiments of Scott Kelso and his group [55,56] that discovered the offline encoding of negation of predicted actions as part of the human mirror neuron system as providing key evidence for the necessary G-T-P logical condition (3) in the cognitive system to achieve the capacity to “think outside the box” and be capable of novelty production. Markose [45] shows how the well-known textbook exposition of Rogers [16] for the G-T-P condition (2) for meta-representation utilizes a 2-place Gödel substitution function. This provides a setting that can incorporate the self and the other as a means of achieving social cognition and social interaction based on the reuse of codes from machine executions of sensorimotor activity by self.

One of the objectives of the paper is to show that there is evidence for an exact same G-T-P mirror/meta recursive machinery that has been found in the Adaptive Immune System (AIS) associated with the so-called Big Bang of Immunology [57]. The evolution of AIS is signposted by the development of the Thymus in the eukaryote lineage since jawed fish some 500 million years ago. The defensive operations of the innate immune system, which can be described as relying on analog tactics (see [58]) were radically reinforced in the AIS by code-centric defenses against digital hacking by non-self antigens. Complex eukaryote development is clearly predicated on the development of the most sophisticated cyber security to overcome the Achilles heel of code-based systems, which are software-related cyber-attacks coming from a plethora of biotic malware. The paradigm shifting nature of general and promiscuous gene expression in the Thymus has been identified as its capacity to “*mirror virtually* (italics added) all tissues of the body” [59]. There is now ample evidence that the Thymus Medulla remarkably expresses copies, in an *offline* environment, of ~85% of the genome [60]. In addition, the most famous case of genomic viral software is the domestication of an ancient transposase that resulted in the recombination-activating genes Rag-1 and Rag-2 [61] for V(D)J combinatorial machinery, which permits diverse concatenations of discrete digital packages of data in gene codes. The extensive thymic self-representation of gene codes has led [62] to call the Thymus as ‘the science of self’ for the purpose of juxtaposing with the V(D)J imprinted T-cell receptors to detect changes in self-codes, crucially but not exclusively, for the identification of the hostile other in the form of non-self antigens. This is considered to be the defining feature of the adaptive immune system (AIS).

In proposing an unitary G-T-P meta/mirror-based recursive machinery in AIS and neuronal social cognition, this paper gives a new explanation for the growing evidence [63], that regulatory factors such as the Interferon Gamma, which is known for identifying friend and foe in the AIS, also has a key role in social behaviors. There is a long legacy, at least since [64], on the immune cognitive system theories of intelligence in which internal self-image is proposed as the basis of the “other”. Many, similar to [65,66,67] and others, make the link between how the immune system became “smart” and the possible similarities in bio-molecular processes underpinning neural activities relating to cognition, communication and signaling, social cognition and even behavioral traits [68]. Ref [69] has conducted an extensive survey of what they term self-referential processing in the brain. Ref [70] goes further and characterizes all biotic elements to be cognitive components imbued with self-referential sensory perception of the “other”. Ref [71] have tried to show how the mechanics of the clonal selection process found in the *Thymic self* carries over to the so called *Brain Self*, though they make no mention of the offline or meta/mirror status of the self-representation. Thus, many of these studies use some metaphors from G-T-P. However, they do not use any of the relevant recursive function machinery, which shows that what seems like disparate aspects of the AIS and MNS are in fact a unitary whole of self-referential information processing in advanced G-T-P style digital systems.

In terms of recent bio-molecular discoveries, Ref [28] discuss how Rag-1 and Rag-2 involved in generating diversity in the immune system via V(D)J recombination are expressed in the Central Nervous System and in olfactory sensory neurons, which are actively involved in experience-mediated neural plasticity. However, as the Central Nervous System is regarded to be immunologically privileged, there is controversy about the extensive studies that show widespread commonality of the biomolecules, initially considered to be the preserve of the immune system, to be in found in healthy brains [72]. These biomolecules relate to diverse sensors/receptors and signaling pathways involving cell to cell synapses to help the organism to maintain homeostatic equilibrium with the somatic self. Despite a growing recognition of the unique self-referential information processing in the *Thymic Self* and *Brain Self*, the discovery in the healthy brain of the vastly polymorphic HLA-DR class of genes relating to Major Histocompatibility Complex (MHC), well known in the AIS for presenting the *Thymic self* and its experiential real time correlates for non-self identification, was considered to be “unexpected” and an anomaly [73]. Further studies [74,75] have shown that neurons in the healthy brain not only normally express MHC I messenger RNA (mRNA), but this expression is vital both in the developing brain and in the mature brain for memory formation and for real time onward synaptic processing for contextual memory, social cognition and novel object recognition. Ref [75] make an interesting observation that it is not that the MHC I deficit mice have impediments to their sensory or motor capabilities as they can see and approach other mice, it is simply that they cannot distinguish between a known mouse and a strange one. Additionally, it has long been known that MHC expression relies on Interferon Gamma both as a trigger and for enhanced pathogen detection in the AIS [76].

**Figure 1:**
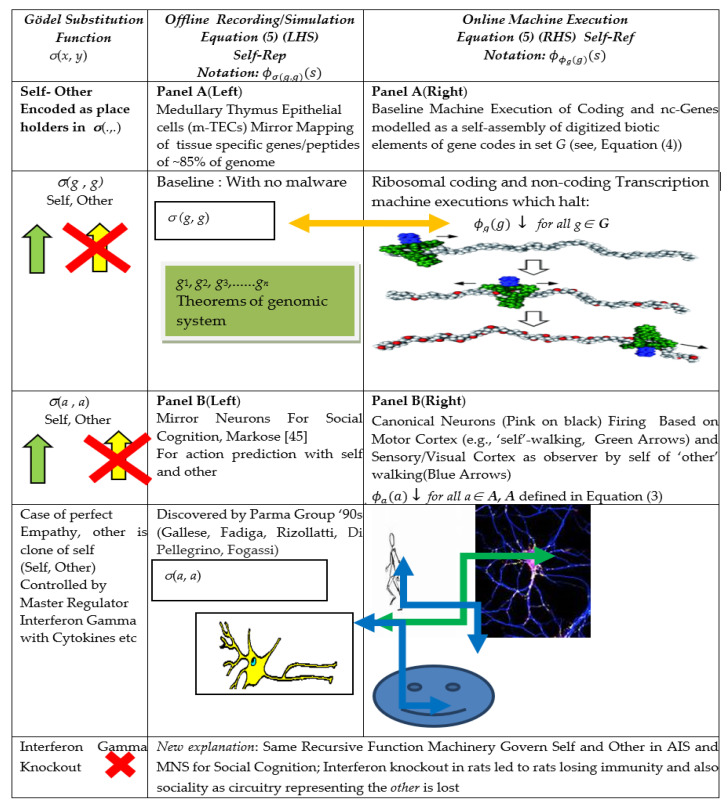
Gödel meta-representation Rogers [16] (Equation (5)) and Mirror Systems in Immuno-Cognitive Systems. Note: *Offline* Mirror Systems in Medulla Thymus (Panel **A** (**Left**)) and *Offline* Cognitive Mirror Neuron System (Panel **B** (**Left**)) and respective Bijective Map of *Online* Gene Transcription (Panel **A** (**Right**)) and *Online* Action Execution in Sensorimotor Cortex (Panel **B** (**Right**)). Interferon Gamma knock out (first left column) affects the 2-place Gödel Substitution function for self and other (*σ*(*g*, *g*) and *σ*(*a*, *a*)) for respective AIS and MNS in *offline* peripheral MHC receptor (see Table 2). This leads to loss of circuitry and hence a blind spot regarding the other in both AIS and MNS, resulting, respectively, in loss of immunity and sociability.

In [63] bio-molecular experiments performed by the Jonathan Kipnis group, it was found that, when the regulatory factor Interferon Gamma is knocked out in rats, the rats not only lost their immune capabilities but also their social skills. As Interferon Gamma has been associated with MHC in the context of the AIS, its role in social cognition was deemed to be ‘unexpected’ by [63]. They offer the following explanation: “Since social behavior is crucial for the survival of a species and aggregation increases the likeliness of spreading pathogens, we hypothesized that there was co-evolutionary pressure to increase an anti-pathogen response as sociability increased, and that the IFN-γ pathway may have influenced this co-evolution.” Ref [68] reiterates a similar view that animals need to balance sociality with the likelihood of acquiring pathogens. Interestingly, these papers do not present evidence that an identical G-T-P meta/mirror-based recursive function machinery underpins cognitive inferences on the other from the vantage of self, both in the MNS for social cognition and in the AIS. An extension of a graph from [45] is given in Figure 1 in the next section to show how the self-referential circuitry on self and the other mediated by MHC and enhanced by Interferon Gamma is integral to the G-T-P model for the mirror systems in both MNS and AIS.

The paper gives the G-T-P based bioinformatics of the AIS that leads to novelty production with hypermutation for new antibodies. Both the V(D)J operations and the somatic hypermutability associated with responses to non-self antigens will be reassessed within the context of G-T-P logic. It must be noted that, as Gödel [35] predates the full developments of machine execution/halting and recursive function theory, many papers that follow the original framework for Gödel incompleteness utilize a formalism for the Gödel sentence (see Endnote [77] and Refs [78,79]) which does not use an explicit mapping, called self-representation, between online machine execution and the copy of the same in the meta offline system. Hence, as in [45], I underscore the importance of using the Rogers [16] meta-representation schema as it allows us to deal with indexes of machines and recursive operations thereof explicitly in their online and offline (meta) domains. Further, I use the Post [37] set theoretic framework of G-T-P formal systems, which is adopted in [16,17]. Here, halting programs of gene codes are “theorems” in the system and negation of these are non-theorems or “forbidden” codes of clones á la Burnet [80], which if they were to run in the system will bring outcomes that are damaging to the host. Again, note Rogers [16] meta-representation schema does not use nomenclature, such as online and offline, with the latter, in particular, being qualified as a virtual simulation and widely referred to as “mirrors” [81] in the above cited AIS/MNS literature. This paper will carefully state how the terms offline/online and meta/mirror apply to the Rogers [16] meta-representation theorem. The proposed powerful organization of digital genomic information in the meta-system of the organism using Post [37] disjoint machine listable or recursively enumerable sets of genomic theorems of gene codes and their non-theorems in the format of Gödel incompleteness has far-reaching implications for genomic information processing. 

Section 2 will set out the formalism and rationale for G-T-P conditions (1) and (2) for Self-Ref and Self-Rep in the immune-cognitive system. For purposes of signposting the contents of the paper, in Section 2, I will also provide a table form summary of the formalism of G-T-P that corresponds to major evolutionary developments and advances in gene and neuro sciences that underpin genomic intelligence. This will be followed by the above-mentioned graphical depiction in Figure 1 of the G-T-P mirror systems at work in the AIS and MNS to familiarize the reader with this framework. In Section 3, a detailed G-T-P model is given of the bioinformatics of the V(D)J recombinase and T-cell training to identify malware function as a fixed point using the Second Recursion Theorem for the G-T-P encoding of the Gödel sentence. What is important to note here is that, for the Gödel sentence relating to a gene code, to self-report that it is under attack, requires that the V(D)J imprints in the T-cell Receptor, produced offline in the Thymus, should match with the record produced by peripheral Major Histocompatibility Receptor (P-MHC) when the non-self antigen attacks online and in real time. This is an original result that throws new light on the genomic intelligence involved in complex interactions involving self and the other and the “smarts” for contextual novelty production. The new G-T-P bioinformatics of AIS and novel anti-body production gives interesting testable implications for COVID-19 pathology. Section 4 will give concluding remarks and outlines further work on implications of G-T-P based genomic information processing, as we have barely scratched the surface.

## 2. G-T-P Conditions and Major Evolutionary Developments in Immuno-Cognitive Systems

As postulated, the driving force behind the unique form of genomic intelligence is the necessity for the genomic system to identify the other, in particular the hostile malware agent that could alter what will be referred to as basal self-codes involved in vital operations governing genomic autonomy/identity and somatic integrity. As discussed in [44,45], this is a digital game with self modelled as the host (*h*) and the hostile other as the parasite (*p*). The necessary G-T-P formalism will also be given. The main hypothesis and the evidence that the immuno-cognitive systems of eukaryotes operate using identical recursive machinery that involves the self and the other will be illustrated in Figure 1.

Table 1 summarizes the three G-T-P conditions in respective panels marked **1**, **2** and **3** that also correspond to major evolutionary developments in the immuno-cognitive systems. In order for Table 1 to show the unitary nature of digital information processing in the AIS and MNS, column 1 gives the necessary G-T-P conditions with the sections of the paper, along with the notation being used and the key equation numbers. Evidence from the literature in gene and neurosciences is also given for each panel of Table 1. Table 1(1a) first cites the evidence for the unique biotic identifiers in the AIS and MNS, which is the starting point of the G-T-P framework, as discussed in Section 2.1. Table 1(1b) gives the basal information that is generated online in halting machine executions internal to an organism, respectively, from gene transcription and translation for somatic morphology and regulation, and the neuronal firings in sensorimotor cortex from self-actions. Table 1(2a) corresponds to the G-T-P condition 2 on offline Self-Representation of the basal information in the *Thymic Self* and in the *Brain Self* for which the former uses the Thymic MHC receptors, while in the latter there is evidence of MHC 1 mRNA. Table 1(2b) outlines the role of the Gödel substitution function to model Self and Other offline in the AIS and the MNS, starting with the benign other as self. Table 1(3a) gives the G-T-P formal conditions for online halting programs “self-repped” into the *Thymic self* and the MNS to be theorems in the system with “forbidden” codes to be non-Theorems. The V(D)J training in AIS T-cell receptors is in relation to the “self-repped” data in Thymic MHCs. Table 1(3a) outlines, what will be shown in Section 3 for the AIS and also in [45] for the MNS, that the G-T-P Self-Rep condition in Equation (5), which includes the Gödel substitution function, is both necessary and sufficient for AIS and MNS to encode the Gödel sentence that effectively permits a self-code to self-report it is under attack. Contextual and code specific novelty production follows from this with evidence, some of it from COVID 19 pathology, that the deficiency of Interferon Gamma I relating to peripheral MHC is to blame for non-production of Covid 19 anti-bodies.

**Table 1:** Gödel-Turing-Post (G-T-P) Conditions for Genomic Intelligence in Immuno-Cognitive Systems for Complex Interactions Involving Self and Other with Contextual Novelty Production.

	G-T-P Conditions	Adaptive Immune System (AIS)	Brain/Neuronal System
**1**	G-T-P Encoded Genomic Basal Information in Fixed Finite Language, Recursive Function Operations on Codes for *Online* Machine Executions: Self–Ref (Self-Reference) and Self-Assembly		
(**1a**) Section 2.1 and Section 2.2	Unique identifiers aka Gödel numbers (gns) from smallest unit of programs/algorithms based on encoded information, Equations (2) and (3) **Notation**: Set ***G*** for gene codes in Equation (2); Set ***A*** for self-actions in Equation (3)	Digitized biotic materials with unique identifiers Transcription Factor Binding Sites and Binding Motifs [82] Blobel (1999) on ‘zip’ codes; Information encoded in biomolecules [29,30]	Unique identifiers for single neurons and neuron-neuron interaction [82,83,84]
**(1b)** Section 2.2	Self–Ref (Online): ***Diag***(*x*) = $\phi_{x}\left(x\right)$, Online halting (↓) self-assembly program *x* instructs machine *ϕ* to run code *x* as its input Equations (4) and (7) [17]	Online Basal Ribosomal and RNA Machine Execution of gene codes as 3D Self Assembly of digitized materials of morphology and regulatory networks ***Diag***(*g*) = $\phi_{g}\left(g\right)$↓, *g ∊ **G***. [85] (p. 30) and [4,5,86]	Online Basal Self-Actions with Canonical Neurons Firing in Sensorimotor Cortex ***Diag***(*a*) = $\phi_{a}\left(a\right)$↓, *a ∊ **A***. [52]
**2**	**G.T.P *Offline* Mirror Systems with One-One Mapping of Online Machine Execution in AIS of Gene Codes and Self Action in MNS: Self–Rep (Self-Representation): Rogers** [16]**, (pp. 202–204)**		
**(2a)** Section 2.3	Self-Rep with *σ*(*g*, *g*) denoting offline virtual simulation for online machine execution $\phi_{g}\left(g\right)$ in Section 2.3, Equation (5) *σ*(.,.) is 2-place Gödel Substitution Function to model Self and Other, respectively, in (*status of self*, *agency of non-self* vis à vis *self*); Basal *σ*(*g*, *g*) shows ‘benign self’ or the absence of non-self antigen	Adaptive Immune System (AIS) Thymic Major Histocompatibility Complex (T-MHC) receptors represent 85% of genome basal self-codes; Equation (7) and Figure 1 (Panel A) and Figure 2 Autoimmune regulator (AIRE) for Thymic MHC expression of Tissue Restricted Antigen (TRA) The other as self-referential projection of self	Mirror neurons in brain have one to one mapping with basal information from online self-actions in sensorimotor cortex in Panel(1b) above; Figure 1(Panel B) Neurons in healthy brain express MHCI mRNA: vital for recordings of experiential data for memory formation on identity of other
**(2b)**	This paper postulates a unitary G-T-P recursive machinery for the immuno-cognitive system, see Figure 1 Refs [63,64,65,66,67,68] on unitary immuno-cognitive systems	Big Bang of Adaptive Immune System 500 million years ago in thymus of jawed fish: [57,59,79] M-TECs mirror the peripheral self [81] *Thymic Self*: [60,62,71]	Mirror Neuron System (MNS) Discovered by Parma Group [47,48,49]: MNS dubbed Great Leap Forward [50] *Brain Self* with MHC I mRNA [73,74,75]
**3**	**G-T-P Formal System of the Other and Novel Hostile Other, Fixed Point of Gödel Liar/Negation as Gödel Sentence (See Section 3)**		
**(3a)**	Domain of ***Diag***(*g*) = $\phi_{g}\left(g\right)$↓, set ***G*** of Theorems (7); Non-theorems are negation or malware operations on gene codes with typical g.ns *f*^¬^◦*g* = *g*^¬^ in set ***G**^¬^*, Equation (8) Non-Theorems involve ‘forbidden’ codes, using a term from [80] G-T-P formal set theory of Post [37], also in [17,38] of creative and productive sets used in Figure 2	V(D)J Simulation of codes of potential non-self antigens in T-cell receptors (TCR) in Thymus Medulla (m-TECs) trained/tested against basal Self-Rep codes in Thymic MHC receptors Partially trained lethal TCR of a novel antigen, Equations (10) and (11), can lead to auto-immune disease Refs [62,87,88], on anticipatory form of V(D)J clone of antigens in TCR in prodigious numbers of between 10^15^–10^30^	The *other* as embodied offline simulation by reusing self-codes [48,49,50,89] RAG-1 and RAG-2 in brain [28] Refs [44,45] show how the 2-place self-other in MNS uses the Gödel substitution function for meta-analyses of second order problems Ref [69] Self-referential processing in brain
**(3b)**	Gödel Sentence in Equation (12) as Fixed Point for novel non-self antigen *f*^¬!^ attacking *g*. G-T-P Second Recursion Theorem of Fixed Point involving malware detection in *peripheral* MHC receptor coinciding with motifs generated in TCRs Section 3.4, Figure 4 Rogers Fixed Point Theorem ([16] Section 11.2) shows that the fixed point of the non-self antigen can be encoded and it will permit a self-code to self-report it is under attack Novelty Production: Section 3.5	Non-Self antigen detection in *peripheral* MHC (P-MHC) receptor has to sync with motifs generated in Thymic TCRs Absence of Type 1 Interferon Gamma at P-MHC leads to failure of somatic hypermutation and novel antibodies produced by B-cells in COVID-19 pathology Refs [76,90] on TCR selection based on affinity or avidity models and self-non self identification in peripheral MHC Refs [91,92,93] on Interferon Gamma I and COVID-19	Scott Kelso and his group [55,56] discovered that offline encoding of negation of predicted actions relating to other is part of human MNS Interferon gamma for non-self in AIS and social cognition [63] Refs [44,45] on arms race in innovation/novelty production

### 2.1. G-T-P Condition (1): Encoding and Recursive Function Operations on Codes

#### Unique Biotic Identifiers and Gödel Numbers

The first G-T-P condition involves the unique digital identifiers for encoded information aka Gödel numbers (g.ns), the recursive function operations on the latter and also their generation and storage (see Table 1(1a)). There is growing evidence of the widespread Gödelization of genomic elements in terms of their unique biotic digital identifiers as Gödel numbers that can be mapped on to the set of integers to denote packets of encoded information. These can operate in a trio of ways: as instructions for machine executions and as their inputs and outputs.

In the work for which he received the Nobel prize in 1999, Günter Blobel discovered unique peptide signals in ribosomal machine outputs. Blobel compared these to postal zip codes that can be read by Signal Recognition Particles so that the ribosomal machine outputs can be directed to specific locations. Unique biotic identifiers in the form of binding motifs have been found to be prolific in transcription factors and their binding sites for associated gene expression in the temporal and spatial development of the multi-cellular tissue-based morphology of eukaryotes and their regulatory networks. Ref [82] found that genes with common DNA “zip codes” can be made to cluster together when called upon by the appropriate transcription binding factor, while those genes that did not have the common zip codes were excluded. Interestingly, [84] first discovered that a large family of genes encoding the proteins protocadherins and isoforms appear to provide the cellular address IDs, similar to barcodes for directing appropriate neuron–neuron interaction. This was later confirmed by [83], that these proteins are expressed in different combinations in individual neurons, thus providing “barcodes” that distinguish one neuron from another and aid in self-avoidance. The extensive use of biotic digital codes as indexes are known to guide messages in the complex RNA gene regulation networks and to help in self/non-self discrimination in the immune system and neural circuitry. While more details are clearly beyond the scope of this paper, examples given here will inform what follows on the significance of the digitization of biotic elements. Further, it follows that machine listable sets of the outputs of genomic codes, where such sets themselves have recursively derived indexes, well known in the work of Post [37], play a vital role in bio-ICT.

### 2.2. G.T-P Gödel Numbering of Basal Information in Gene Codes and Sensorimotor Cortex

In view of digitized materials being represented by integers a.k.a. Gödel numbers, operations involving these can likewise be encoded and represented in a unique way. Hence, genomic operations on encoded information belong to the class of recursive or computable functions. The latter are number theoretic functions, *f: ℵ→ℵ*, where *ℵ* is the set of all integers and constitute the domain (inputs) and range (outputs) of these code-based functions (see [17] and [94]). As computable functions have to be executed using a program or algorithm, such functions have a standard notation [17] that take the following form with the index or g.n of the program that computes it, given as a subscript of the computable function:

(1) $$f(y)\;≅\phi_{x}\left(y\right)\;=\;q.$$

That is, the value of a computable function *f*(*y*) when computed using the program or Turing Machine (T.M.) with index *x* on input *y* is equal to the output given by an integer *q*, $\phi_{x}\left(y\right)$*= q* if $\phi_{x}\left(y\right)$ is defined or halts (denoted as $\phi_{x}\left(y\right)$↓), or else the function *f*(*y*) is undefined (~) if *ϕ_x_*(*y*) does not halt (denoted as $\phi_{x}\left(y\right)$↑). A computable function $\phi_{x}\left(y\right)$ is defined to be total if it halts on all *y ∊ ℵ*. A partial computable function, $\phi_{x}\left(y\right)$, does not halt on all *y ∊ ℵ*.

The Turing Machine models of bio-molecular computing evidenced in ribosomal RNA are well known [4,9,10,95] and the literature abounds with many metaphors regarding the digitization of inheritable information. However, what is important to note is the following 21st century nomenclature of Gershenfeld [85] (p. 30) which underscores how the RNA machine execution of gene codes are programmed as 3D self-assembly machines (see [4,85,96,97]). The model of 3D self-assembly here is of a gene code as a program, *g*, which instructs the RNA machine, which is either a ribosome or other RNA transcripts to run itself viz. the same code, *g*, is executed as a series of subprograms as inputs. These inputs can include the transfer RNA (tRNA) to assemble the biotic materials, such as amino acids, which are then effectively “printed” as other outputs. The latter can be viewed as digitized materials for somatic morphology (tissues and organs) and their regulatory structures. Protein coding genes are famously known to have stop codons of UAA, UAG, or UGA, which mark the completion of the program. The density and status of stop codons in non-coding genes, especially, in so called long non-coding RNAs (lncRNA) and circular RNAs and also the functionality of their outputs, often taking the form of regulatory micro-peptides, continue to be a matter of intense research [98]. For the purposes of this paper, all gene codes whether protein coding or non-coding will be deemed to belong to an important class of halting self-assembly machines in the above sense. These have a natural status in generating basal building blocks in genomic systems and will be shown to have special significance in G-T-P computation theory, where they are referred to as online Self-Ref (Self-Reference) or ***Diag***(.) operations (Table 1(1b)). The formalism for this is developed below.

Using the well-known system of Gödel numbers (g.ns), integers can uniquely identify gene codes based on the near universal alphabet of the genome. The set of gene codes representing self-assembly programs that are tissue specific and accompanied by their cognate regulatory networks arising, respectively, from protein coding and non-coding (nc) ones is denoted as:

***G*** = {*g_1_, g_2_, ……, g#*}. (2)

Any gene code will be generically denoted as *g*, and # denotes some finite cardinal number. The digital encoding of the finite set of states under which the genes are transcribed is denoted by, ***S***, with *s ∊ **S*** is an element in a finite and countable set of states and other archival information (see [99]). Likewise, the set of online action related data from the canonical neuronal firings in the sensorimotor cortex when action is undertaken by self will be denoted by set ***A***,

***A*** = {*a*_1_, *a*_2_, ……, *a#*}. (3)

The significance of basal information in set ***A*** in terms of what [52] call firing of canonical neurons in the sensory-visual and motor cortex for social cognition was recently cited by [100] as “a large, complex and ancient set of Bayesian priors (visual, sensory, motor) that constrain inference in any mammalian brain, and are equally operative in the human brain” (see [44,101]). 

In the following section, while the narrative is primarily in terms of the tissue specific gene codes in set ***G*** in (2) for the mirror system in the adaptive immune system, as the graphics in Figure 1(Panel B) show, on replacing this by set ***A*** in (3), we have an identical mirror system for the cognitive mirror neuron system.

#### Online Self-Assembly/Self-Ref Machinery

In order to represent the online self-assembly model of the ribosomal RNA or the non-protein coding transcription machinery, the following notation from Rogers [16] is used to represent the online machine execution of gene codes *g ∊ **G***:

(4) $$\phi_{\phi_{g}\left(g\right)}\left(s\right)=q,\;Diag(g)\;=\;\phi_{g}\left(g\right)↓:\;\mathrm{Self}-\mathrm{Ref}.$$

Here, the $\phi_{g}\left(g\right)$ in the subscript of the recursive function ϕ that outputs *q* in (4) underscores the online self-assembly or Self-Ref operation, denoted as ***Diag***(*g*) = $\phi_{g}\left(g\right)$, such that a gene encodes a program *g* that instructs a machine $\phi_{g}\left(.\right)$ to run *g.* The output of $\phi_{g}\left(g\right)$ becomes the program for further computation which results in the output *q* in (4). As indicated, the program *g* that instigates the self-assembly process in Equation (4) halts, $\phi_{g}\left(g\right)$↓, and outputs digitized materials such as proteins that have embedded in them programs for further steps in the assembly process which include zip codes for their location. The output *q* in Equation (4) that follows from the full transcription/translation process signifies, respectively, a somatic tissue in the case of a coding gene, or an RNA regulatory phenotype based on a non-coding (nc) gene.

The strenuous proofreading aspects discovered [11,12] in the ribosomal self-assembly procedures in Equation (4) where errors in the inputs and outputs are controlled, can be taken as the starting point for defending the primacy of the code that leads to integrity of the somatic and regulatory outputs represented by *q*. Indeed, this digitized online basal information in the respective immuno–cognitive systems given in sets ***G*** in (2) and ***A*** in (3) will be shown to be “theorems” of the systems and define the objective of the genomic game as one in which the host has to retain the genomic identity and somatic integrity of the basal codes in terms of the phenotypes or the outputs generated from them.

### 2.3. G-T-P Mirror/Meta Condition 2 and Evidence from Genomic Evolution of Self-Rep 

The identification of pathogens or of non-self other is part of prokaryote cell biology and of a highly sophisticated Innate Immune System. These predate the developments in the Thymus associated with the Big Bang of the Adaptive Immune System and also in the so-called Great Leap Forward that [50] associates with the discovery of the Mirror Neuron System by the Parma Group in the 1990s. The paradigm shift with the extensive gene expression identified in medullary thymic epithelial (m-TEC) cells, “of virtually all tissues of the body, irrespective of developmental or spatio-temporal expression patterns” [59], had led [81] to state that “m-TECs may indeed represent an immunological homunculus, in that they mirror and anticipate the peripheral self”. So, why can the information processing of the AIS and the Mirror Neuron System and not that of the Innate Immune System be characterized by the G-T-P mirror/meta conditions for Self-Rep (Self-Representation)?

For this, note the famous *offline* Gödel Meta-Representation system that maps 1–1 from the basal online machine executed data in the format from Rogers [16] (pp. 202–204):

(5) $$\phi_{\sigma \left(g,g\right)}\left(s\right)≅\;\phi_{\phi_{g}\left(g\right)}\left(s\right)=q,\;iff\;\mathrm{Diag}(g)\;=\;\phi_{g}\left(g\right)↓:\;\mathrm{Self}-\mathrm{Rep}.$$

Here, *σ*(*g*, *g*) in the subscript on the LHS in (5) is the index for the program that *records* or *copies* in the offline meta-system the corresponding online machine execution, denoted by the subscript $\phi_{g}\left(g\right)$ on RHS of (5) for the “self” gene codes *g*, *g* ∊ ***G*** in (2), that produces output *q* for the organism. 

This warrants the moniker of Self-Rep (Self Representation) of Hofstader [40]. The Self-Rep scheme in Equation (5) highlights the two-step procedure mentioned in [46] on the underlying operation of self-reference, ***Diag***(*g*) on the RHS of (5) and the copy of the same on the LHS of (5). Meta-analyses of software based alterations to basal codes, as will be seen, involve virtual recursive function operations on *σ*(*g*, *g*). The Rogers [16] meta-representation schema in (5) and the graphics in Figure 1 will be used to show that an identical recursive machinery can be conjectured to be at work in both the Adaptive Immune System (Figure 1(Panel A)) and the Mirror Neuron System (Figure 1(Panel B)).

Specifically, the LHS function *σ(g*, *g)* in (5), also shown in Figure 1(Panel A), modelled along the lines of the Gödel two-place substitution function [16], has the feature that it names or “signifies” in the off-line recording in the Thymus Medulla epithelial cells, m-TECs, the following information: *σ*(*g*, *g*) is the total computable index function for program for the self-assembly machine ***Diag***(***g***) = *ϕ_g_*(*g*) such that if and only if (*iff* in Equation (5)) it halts and generates an output, that is used for further computation to produce the final output *q* viz. $\phi_{\phi_{g}\left(g\right)}\left(s\right)=q,$ then meta/mirror system also faithfully predicts the outcome is *q.* As in general, ***Diag***(*x*) = $\phi_{x}\left(x\right)$, *x* ∉ ***G*** may not halt, the corresponding *σ*(*x*, *x*) computation on LHS of (5), will not halt.

In Greenen [62], two processes are given for the presentation of the information in self-gene codes of the host in the m-TECs. The primary approach relies on thymic extraction of peptides from most self-gene codes to be displayed via the Thymic Major Histocompatability Complex (T-MHC). Greenen [62] notes these self-gene proteins “are processed in TECs to form an array representing self, which are eventually presented in the context of MHC molecules” so that the generation of adaptive immune system repertoire to protect self-genes are “educated on self”. The other route for the presentation of so-called Tissue Restrictive Antigens (TRA) in the thymic MHC relies on the autoimmune regulator (AIRE) [60], and related factors. The consensus is that the mTECs are specialized to express a highly diverse set of genes, essentially representing all tissues of the body, also referred to as the peripheral self [81]. The latter constitutes the somatic morphology of the organism, arising from the online halting self-assembly machines for gene codes *g* ∊ ***G*** in (2) on the RHS in (5) and in Figure 1(Panel A), which depicts ribosomal machine execution. As not all *g* ∊ ***G*** in (2) have a self-representation in the m-TECs, it is useful to define a set ***G^#^*** ⊂ ***G*** that are “Self-Repped” in the m-TECs.

In general, in order to identify the non-self other, based on the thymic MHC self-representation of what [62] calls the “benign” self, which entails halting ***Diag***(*g*) = $\phi_{g}\left(g\right)$, the information processing in the meta system characterized by the two place-holders in the Gödel substitution function *σ*(*g*, *g*) on LHS of Equation (5) is conjectured to take the following form. From the perspective of the host, the first entry below in *σ*(., *.*) relates to self directed actions and the second entry relates to the agency of non-self, vis à vis self:

*σ*(*self actions, agency of non-self vis* à *vis self-actions*).

Thus, for the AIS, in the *σ*(*g*, *g*) notation in (5), in the first place from the left, is the record of host’s gene code and an identical *g* in the second place implies that the host has identified that there has been no alteration of this gene code by the non-self antigen or pathogen, aka Liar/hacker. In other words, the agency of the other is calibrated self-referentially, viz. in terms of self-codes. This two-place notation of the self and the other in the meta representation *σ*(*g*, *g*) for the AIS is shown in Figure 1(Panel A) in the first column from the left.

In the case of the mirror neuron system (MNS) being a meta-representation, as in (5), of the sensorimotor cortex, involves neuronal codes pertaining to self-actions, *a* ∊ ***A*** in (3). On changing the notation in the self-representation schema in (5) from *g ∊ **G*** to *a* ∊ ***A*** in (3), following [49,89] of MNS fame, the LHS of (5) is characterized as *embodied off line simulation* of self-actions, *a* ∊ ***A*** in (3), that arise from canonical neurons that fire with action-related motor activity by self [52]. Figure 1(Panel B) shows the synchrony in the mapping between the canonical neuron in black that fires on RHS (with the motor activity of walking by self as an example) with the mirror neuron (in pink) firing on LHS. The host observing another conspecific walk triggers the same mirror neuron as with self-action for this. Assigning the same second place holder to the other, as in the AIS, the meta function, *σ*(*a*, *a*), in the MNS permits an identical action prediction in the other, which is self-referentially accomplished by the reuse of self-codes. As indicated in the Introduction and Table 1 (2b) and (3a), analogous to the role of Thymic MHC for the self-representation of basal self-gene activity in (5), it can be conjectured that MHC1 expressed in the healthy brain maps basal self-activity in the sensorimotor cortex to an offline mirror domain during neo-natal brain development and also primes, with the aid of Interferon Gamma, the synaptic circuitry during real time experiential sensory–visual activity regarding the other [73,74,75].

The unifying building block in the form of G-T-P meta-representation in (5), illustrated in Figure 1 (first column on left), for genomic intelligence in immuno-cognitive systems enables the host to make inferences about the other in the AIS or MNS self-referentially from the recording of codes involved in self action, respectively, given in set ***G*** and set ***A***. This yields a different explanation from that given by Filiano et al. [63] for the finding that, when master regulator Interferon Gamma, known to facilitate the identification of the other, is knocked out in rats, they lost both their immunity and their social skills. The knockout of Interferon Gamma, shown in Figure 1 as red crosses on the circuitry on the place holder for the other in the Gödel substitution function in recursive meta/mirror machinery for the immuno-neural system, results in a cognitive black out in social cognition in the MNS and an inability to identify the agency of the non-self antigen in the AIS. In contrast, Filiano et al. [63] give a “just-so” story: “Since social behavior is crucial for the survival (and since) aggregation increases likeliness of spreading pathogens, so as immunity fails, so does sociability.”

There is considerable literature on the knockout of the auto-immune regulator AIRE, which leads to “blindness” with regard to certain self-gene codes that fail to get “Self-Repped” and so lead to autoimmune pathologies [62]. There is much less understanding of the unitary nature of the Self-Rep recursive function machinery of genomic intelligence in *both* the AIS and social cognition in the MNS. Hence, the role of MHC related genes and Interferon Gamma that regulate a common circuitry in the AIS and MNS for the self-other nexus, where the other is based on self-referential projections of self-codes, is overlooked.

The premise here is that, unless there is an exhaustive listing of basal gene codes, as in the genomic m-TECs, and in the cognitive mirror neuron system of self-codes for sensorimotor activity, the anticipation of algorithmic alterations in the basal codes by the other, viz. malware detection in the case of the AIS in m-TECs and action prediction and intentionality of the other in cognitive systems, is not feasible. Table 2 gives the two main time dimensions over which the AIS and MNS have to process the internal basal Self-Repped information with their respective real time external stimuli. The non-self other is external to the host agent and offline peripheral receptors record external stimuli in real time. The key bridging mechanism for self to anticipate the other, is the two place Gödel substitution function *σ*(.,.) (Table 2 Col.3), which is used to conduct offline simulations from the basal Self-Rep information (Table 2 Col. 2). As discussed in [44,45] it is useful to consider the self-other nexus to be part of a game, and self will be given an index *h* (host) and the other has index *p* (parasite). The hostile other is a special case of other’s actions, given as *f_p_*, which appears online (Table 2 Col. 5). In the case of the MNS, there is evidence (summarized in Table 1(3a) and illustrated in Figure 1(Panel B) and Row 2 in Table 2 Red Arrow) that experientially derived visual–sensory data on conspecific actions is mapped to the appropriate basal self-codes for the automatic action prediction of other.

Further information processing using the Gödel substitution function *σ*(*a*, *a*) with regard to the other in the MNS, has been modelled in [45] with special emphasis on the negation operator involved in the hostile other. As noted, there is evidence from experiments of Scott Kelso and his group [55,56] that the offline encoding of negation of predicted actions are part of the mirror neuron system. In the case of the AIS, the non-self other is directly a software agent and its activity is a recursive function operation online on heathy self-codes of the host, Table 2 Row 1 Col. 5. Thus, the moot point of self-referential information processing regarding the other in the AIS and MNS, as highlighted in Table 2, is that the offline Self-Repped basal self codes in Equation (5) (Table 2 Col. 2) are the starting points for all subsequent genomic intelligence. 

**Table 2:**
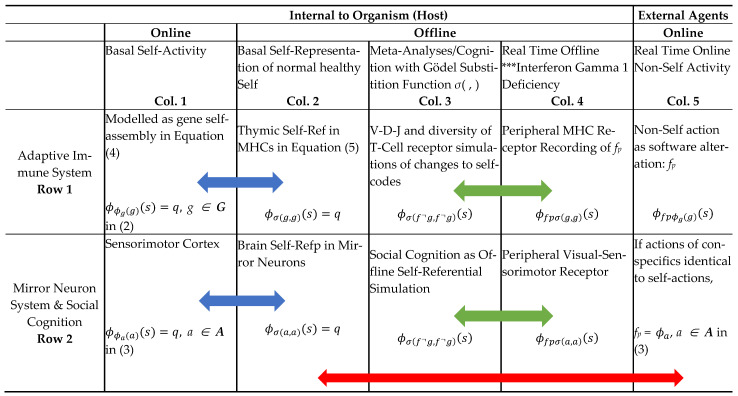
Two Main Time Dimensions for Online/Offline Information Processing in AIS and MNS: Basal and Internal to Host Self-Rep (Cols 1,2) and Real Time (Cols. 4 and 5) with External Stimuli. Note, Interferon Gamma 1 Deficiency will cause “blind spots” in the Peripheral MHC receptors to other *f_p_* actions in relation to self (marked with asterisks in Col 4); Blue Arrows show the offline Self-Rep of basal online machine executions; Red Arrow show the host observation of conspecific actions that are identical to self-actions leads to action prediction; Green Arrows show how V(D)J and simulation with Gödel substitution function will provide the fixed point to identify external changes to self-codes.

In preparation for Section 3, which develops the primary objective of the AIS in terms of identifying the non-self antigen relating to G-T-P condition (3) on incorporating the hostile other as a negation operator on basal self-codes, three further points are made here on the meta-representation schema in Equation (5). Rogers [16] characterizes the *σ*(*g*, *g*) Gödel substitution function as a powerful tool of meta analyses in that the host can generate different “propositions” to signify what is happening online ((RHS) of (5)) as it cycles through the tuple (*g*, *g*) using integers that exceed *g* ∊ ***G***. As will be shown, this can explore the table of recursive functions given in [17] and adapted in Figure 3 below. The AIS effectively has to track and identify changes in basal gene codes brought about by a biotic digital agent, viz a recursive function. For this, the Rogers [16] meta-representation schema in (5) provides an easy way to determine the fixed point of a (total) recursive function, which, in the case of a “negator” antigen, denoted as $f^{\neg }$, that operates on the baseline *σ*(*g*, *g*), via the Second Recursion Theorem: $\phi_{f^{\neg }\sigma \left(g\neg ,g\neg \right)}\left(s\right)≅\;\phi_{\sigma \left(\;g\neg ,g\neg \right)}\left(s\right)$, here g.n $f^{\neg }$◦*g = g*^¬^, and *σ*(*g*^¬^, *g*^¬^) is the fixed point of $f^{\neg }$ [17].

Finally, the meta-representation framework in (5) makes it possible to keep track of the indexes for the programs involved both in the online and offline (meta) domains. These can have different time dimensions shown in Table 2 as operations that occur in real time (Cols. 4 and 5) and those that are called “basal” (Table 2 (Cols 1 and 2)) having occurred in the early development of the host. Offline meta-analysis in Table 2 Col. 3 can include calculations that can be in real time or prepared in advance and, hence, anticipatory. The reason these different time domains are important for the immuno-cognitive system is because the antigen attack, $f^{\neg }$, happens online as a real time machine execution on ***Diag***(*g*) = *ϕ_g_*(*g*), relating to some specific tissues or regulatory factors in what immunologists call the periphery. The offline meta recording domain for the real time experience of the $f^{\neg }$ inflicted change in basal self-code, viz. $f^{\neg }$***Diag***(*g*), is now in the peripheral histocompatibility complex (P-MHC) found in non-thymic peripherical cells. As we will see, the failure to update the record of the real time attack $f^{\neg }$***Diag***(*g*) in the P-MHC in order to be part of the explicit meta representation using the Second Recursion Theorem of the fixed point for $f^{\neg }$, as in the left hand side of $\phi_{f^{\neg }\sigma \left(g\neg ,g\neg \right)}\left(s\right)≅\;\phi_{\sigma \left(g\neg ,g\neg \right)}\left(s\right)$, could be fatal. Further, the updated P-MHC record $f^{\neg }$*σ*(*g*^¬^, *g^¬^*) has to coincide with the index for the fixed point $f^{\neg }$ for on the right-hand side viz. *σ*(*g*^¬^, *g^¬^*), which has to be generated in offline “theoretical” cloning of non-self antigens in the m-TEC trained T cell receptors in relation to the basal self-codes expressed there. How does m-TEC TCR training and selection successfully generate such *σ*(*g*^¬^, *g*^¬^) indexes as fixed points of potential online antigen attacks? Section 3 will show how the G-T-P logic behind the selection of V(D)J generated motifs in T-cell receptors are geared toward the online and real time identification of non-self antigen attacks with the updates in the peripheral MHC meta index. This results in the successful encoding of the Gödel sentence in the above fixed point. Without this, the production of novel antibodies in response to an attack in the periphery from a novel antigen will not be possible. Needless to say, the evolution of genomic intelligence in the AIS and its exaptation in the MNS has rarely, if ever, been framed as emanating from a cybersecurity problem arising from a digital genome and the digital game that has ensued between the host and digital biotic parasites.

## 3. G-T-P Bio-Informatics for V(D)J Recombination and T-Cell Training for Non-Self Antigen Detection

### 3.1. Horizon Scanning and Astronomic Numbers in AIS

At least three sets of statements stand out in the extensive evidence marshalled in the numerous studies on the revolutionary nature of the RAG activated V(D)J recombinations in the T-cell receptors (TCRs):

1. In having mirrored/expressed ~85% of gene codes in m-TECs, the V(D)J recombinations generate putative clones of non-self antigens in relation to these gene codes.
2. This provides “an anticipatory system of defense” [87] of prodigious capacity. Ref [88] state that the capacity of the AIS for “somatic generation of immune recognition motifs of a system of practically unlimited (open-ended) information capacity” with orders of magnitude of “αβT cell receptors to be around 10^15^ to 10^20^ with such levels of diversity in a single individual that exceeds the size of the entire germline genome by several orders of magnitude.” [62] gives an even higher number for the V(D)J generated “individual antigen receptors computed to be approximately 10^30^”.
3. Ref [102] (Chapter 8) ask the following question in the context of Bio-Inspired Computing and Cyber Security:

“*Any paradigm for computer security that is based on the differentiation of self from non-self must imply some operational definition of self that represents normal and benign operation. It is clear that a good definition is matched to the signature of the threat being defended against, and hence the designer must be able to answer the question, “How would I know my system were under attack?*”

(Ibid, p. 263)

In the context of decentralized systems, one can add here that the evidence of attack should be made apparent at the level of each gene code from the set of basal codes of set ***G***. The Self-Rep *σ*(*g*, *g*) in Equation (5) for the halting online self-assembly ***Diag***(*g*) yields the “*operational definition of self that represents normal and benign operation”*. Further, each gene code in Equation (2) should be able to self-report that it is under attack if that is the case. As will be seen, only this can trigger specific and novel antibody production.

Pushing the agenda that the G-T-P framework provides a unifying set of answers to the above, I start with how computable operations that can be undertaken by either the host or the parasite using total computable functions justifies the astronomic numbers for potential new algorithms. Formally stated, when modelled as a digital game, the strategy functions for the host and the parasite *f_i_*, *i∈* (*h*, *p*) that can alter the basal information in sets ***G*** and ***A*** (see [44,45,103,104]) are total computable functions, such that the *g*.ns of *f_i_*, *i ∈* (*h*, *p*) are contained in set ***ℜ***,

***ℜ*** = {*m* | *f_i_ = ϕ_m_, ϕ_m_ is total computable*}. (6)

The set ***ℜ*** of all total computable functions, is not recursively enumerable or capable of being listed by an algorithm. The proof of this is standard [17] (p. 127). In the case of the AIS, representing known members of set ***ℜ*** to be given in set G** where G** includes *known* non-self antigen codes and auto-immune codes along with self-gene codes in ***G***, the g.ns in set ***ℜ***−G** present non-enumerable infinite numbers of ways for new technologies or phenotypes that can be formed and, hence, also the potential malware alterations to gene codes. A potential novel negation/malware function is denoted by *f_p_*^¬!^ = *ϕ_m_*, *m ∈* ***ℜ***−G**. The subscript *p* in *f_p_*^¬!^ signifies that a non-self antigen and the exclamation mark in superscript underscores the surprise entailed in the novel non-self antigen. As will be seen, the nature of set ***ℜ*** accounts for the astronomic numbers in the speculative search for the codes for the algorithms for novel malware, evidenced on a daily basis in the V(D)J generation of motifs in T-cell receptors. 

### 3.2. Halting Self-Assembly Gene Codes and Forbidden Codes of Antigens in G-T-P Formal System

The property of G-T-P relating to Self-Ref and the set of halting self-assembly gene codes mirrored in m-TECs in (5) and their known set of antigens can be viewed as a textbook case of the set theory of G-T-P formal systems first developed in Post [37].

**Figure 2:**
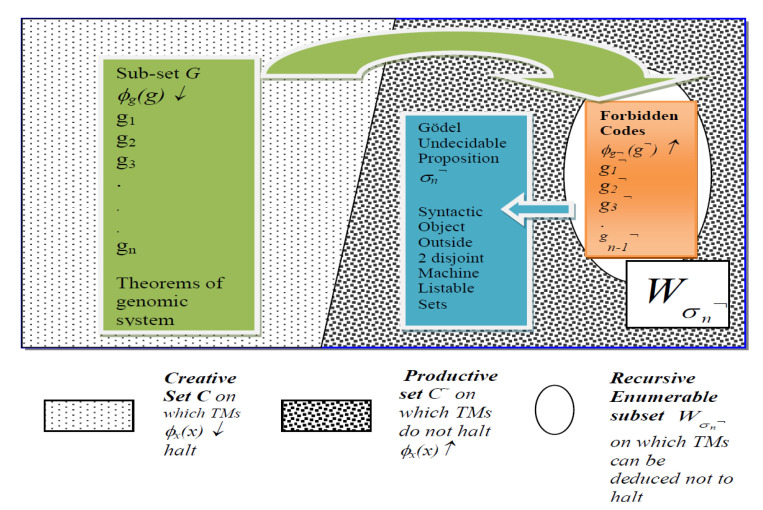
Gödel Incompleteness Result in Miniature: Illustration of Self-Representation in Thymus Medulla of set G* of Gene/Self Codes that are Theorems in Genomic System (LHS, Green) and Set of “Forbidden” Codes of Non-Theorems of known non-self antigens and autoimmune attacks (RHS, Orange). Gödel undecidable proposition *σ_n_*^¬^ lies outside the two disjoint listable sets G* and $W_{\sigma_{n}\neg }$, such that *σ_n_*^¬^ *∉ **C*** ∪$\;W_{\sigma_{n}\neg }$, G* ⊂ ***C***. Note *τ*(*g_n_*^¬^)* = σ_n_*^¬^ will be shown to be the index of clone generated by T-cell Receptors for the ***novel*** *f*^¬!^ antigen that attacks gene code *g_n_*.

Figure 2 shows that the G-T-P formal system is composed of Theorems where constructive proof is a halting computation and non-theorems have no proof, and hence no halting computation. What is important to note is the set G* in green in Figure 2, defined as the domain of the halting self-assembly function ***Diag***(*g*) *=* $\phi_{g}\left(g\right)$*↓* of gene codes in (2) can be shown to be the subset of the archetypal creative set ***C*** (see [17] (p. 133) and [105]). The latter is a machine listable set of all self-referential machine calculations that halt, denoted as *ϕ_x_*(*x*)↓ for any *x ∈ ℵ,* where *ℵ* is the set of all integers. Set ***C*** is central to Post [37] set theoretic proofs for Gödel incompleteness and the classic Post [37] set theory of creative and productive sets of codes. Figure 2 gives what [17] (p. 148) calls the miniature form of the Gödel Incompleteness Theorem, adapted for our case.

Thus, set G^*^ is formally defined in (7) as the domain of halting self-assembly operations ***Diag***(*g*)=$\phi_{g}\left(g\right)$*↓* for the gene codes in set ***G^#^*** that are self-represented via the thymic MHCs in the m-TECs:

(7) $$G^{*}\;=\;\{g|Diag(g)\;=\;\phi_{g}\left(g\right)↓;g\;∊\;W_{g},for\;g\;∊\;G^{\#}\},\;G^{*}\;\subset \;G\;\subset \;C.$$

Figure 2 illustrates how set G*, which is self-represented, as in (5) in the Thymus Medulla constitutes a very large number, but not all, of the basal gene codes of set ***G*** of the halting self-assembly RNA machines (left hand side of Figure 2). Set G* is a subset of the Post [37] creative set ***C*** and hence is identical to a listing of theorems in a formal system. The listing of non-theorems of the system, which are the so called “forbidden” codes using a term from [80], are those codes that should not be executed online in the genomic system, as they will produce outcomes that are antithetical to the original gene codes or theorems of the genomic system. A halting machine execution of *g*^¬^, which denotes a non-theorem, will imply the destruction of specific somatic/tissue of *g ∊ **G*** and the phenotype associated with it. Hence, the forbidden codes, belong to the set of non-halting codes denoted by ***G*^¬^**, disjoint from the gene codes or theorems of the system.

(8) $$G^{\neg }\;=\;\;W_{\sigma_{n}\neg }\;=\;\{g^{\neg }|\phi_{g\neg }\left(g\neg \right)\;↑;\;\mathrm{iff}\;g\;\in \;W_{g},\;\phi_{g}\left(g\right))↓\},\;W_{\sigma_{n}\neg }\subset C^{\neg }.$$

Note that the symbol “¬” denotes negation or ‘not’. The important point is that ***G*^¬^** is a machine listable set ***G*^¬^** = $W_{\sigma_{n}\neg }$ and is a subset of the set $C^{\neg }$ on which ***Diag***(.) machines do not halt**** (see Endnote [105] and the set colored orange in Figure 2). However, $W_{\sigma_{n}\neg }$ has the property that it is only listable up to a point, as will be explained below. What does ***G*^¬^** = $W_{\sigma_{n}\neg }$ contain? It contains codes denoted as *g_n_*^¬^ that are generated in the domain of the fixed point of the *f*^¬^ non-self antigen and autoimmune function, that has altered the basal gene code *g_n_ ∈* ***G*** with the g.n *f*^¬^◦*g_n_* = *g_n_*^¬^ in such a way that the output *q* of $\phi_{\phi_{g}\left(g\right)}\left(s\right)$= *q* of the ***Diag****(g_n_*) program has been “negated” to produce *q*^¬^.

As will be seen, the problem of identifying *g_n_*^¬^ as a “forbidden code”, as in (8), when such codes are generated from a novel software based non-self antigen *f_p_* ^¬!^ that can attack [106] the gene code *g_n_* and bring about the negation of its ***Diag***(*g_n_*) output *q* as shown in (9), is that there is no way to produce an algorithmic listing of this in advance within set ***G*^¬^**.

(9) $$\phi_{f_{p}\neg !Diag\left(g_{n}\right)}\left(s\right)=\;\neg \;\phi_{\phi_{g}\left(g\right)}\left(s\right)=\;q^{\neg }\mathrm{Iff}\;\phi_{\phi_{g}\left(g\right)}\left(s\right)=q,\;\mathrm{Negator}\;\mathrm{Malware}$$

Famously a syntactic encoding of the fixed point of *f_p_*^¬!^, in the form of the Gödel sentence for *f*^¬!^ in relation to the gene code *g_n_*, can be shown to be generated by the genomic system. Further, the index *τ*(*g_n_*^¬^) = *σ_n_*^¬^ generated from the Gödel sentence for *f_p_*^¬!^ in relation to the gene code *g_n_* provides a constructive witness for the Gödel incompleteness of genomic system. This is possible *if and only if* (*iff)* genomic information is organized in a consistent formal system, as in Figure 2. As explained in [45] (Lemma 3), the index *σ_n_*^¬^ for the set ***G*^¬^** = $W_{\sigma_{n}\neg }$ entails a recursive enumeration function *τ*(*g_n_*^¬^) = *σ_n_^¬^*, such that nth element *g_n_*^¬^, indexed as *σ_n_^¬^*, can only be added to the machine listable set $W_{\sigma_{n}\neg }$, but cannot belong to $W_{\sigma_{n}\neg }$. Thus, as shown in Figure 2, *σ_n_^¬^* ∉ G^*^ ∪ $W_{\sigma_{n}\neg }$ is shown to be a witness of a novel forbidden code, which is an undecidable proposition in the genomic formal system in that it can be constructively produced as proof that it cannot be recursively enumerated by the system. 

How can a digital genomic system identify that a specific gene code of a tissue (say the respiratory one in view of COVID-19) has been attacked online and in real time and also know the precise code of the attacker? If the pathogen is one already in the set $W_{\sigma_{n}\neg }$, the host can trivially identify this recursively and use a known antibody. The identification of a novel *f_p_^¬^*^!^ non-self antigen and to respond with a novel antibody to neutralize the *f_p_^¬^*^!^, though ubiquitous in genomic systems of eukaryotes, requires the machinery for Gödel incompleteness to explain this, and note this exceeds standard game theory (see [44,45] and Endnote [103]). Section 3.3 and Section 3.4 will explain this in terms of the AIS and also what has been observed in pathology and recovery in individuals with COVID-19.

### 3.3. Information Processing in G-T-P Meta Systems for V(D)J: Positive Selection of T Cell Receptors

**Figure 3:**
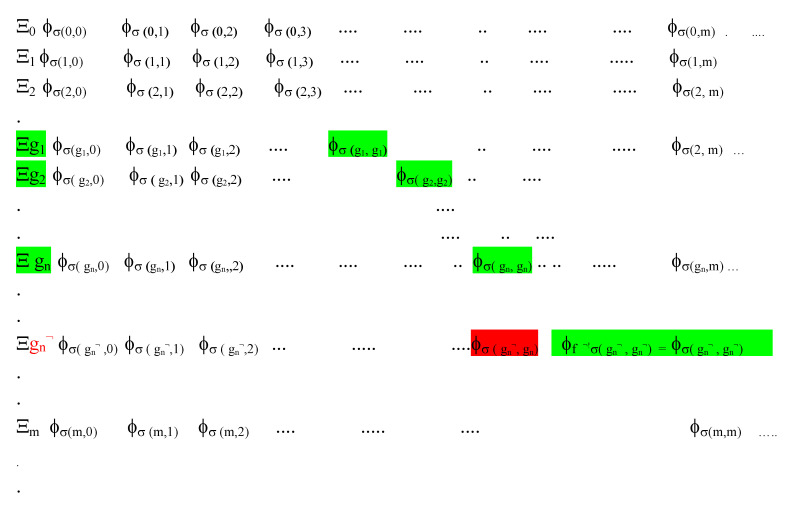
Table **Ξ**, Adapted from [17] sets out the G-T-P model for T-cell Training as V(D)J generated codes/motifs simulate software-based or recursive function alterations to “benign” Self-Rep data given by the green diagonal elements *σ*(*g*, *g*) for halting online basal ***Diag***(*g*) =$\;\phi_{g}\left(g\right)$ genomic operations. V(D)J T-cell receptors simulate applications of total recursive functions *f* on *σ*(*g*, *g*) with an altered gene code generically denoted with a g.n *f◦g* which indexes a new row in Table **Ξ** beyond the ones with index *g*. The g.n for the negator malware *f*^¬^*◦g_n_* is marked in row *g_n_*^¬^ and a T-cell Receptor with the motif *σ*(*g_n_*^¬^, *g_n_*) marked in red will be shown to be dangerous in Equation (10), as it is indicative of auto-immune pathology. The Gödel sentence for {*f*^¬^, *g_n_*} is given as ϕ_*f*^¬!^*σ*(*g_n_*^¬^ , *g_n_*^¬^)_ = ϕ_*σ*(*g_n_*^¬^ , *g_n_*^¬^)_ where the fixed point motifs *σ(g_n_*^¬^, *g_n_*^¬^) in TCRs will anticipate potential attacks of *f*^¬^ in the periphery without harming self-codes.

In order to detect alterations to gene codes that can be done by a non-enumerable infinite set of novel pathogens *f_p_^¬^*^!^, it is important to understand how a record of the Self-Rep data in (5) for the AIS can be embedded in meta information of G-T-P system for all the recursive functions (both partial and total). For this, there is a standard countably infinite Table **Ξ** for recursive functions (Figure 3 adapted from [17]) indexed by g.ns along the rows of the matrix. Of these, only the subset shown as the green elements *σ*(*g*, *g*) along the diagonal of Table **Ξ** representing the halting self-assembly executions of the gene codes in set *g*  ∈  G* of Equation (7) are recorded in the thymic MHCs. There is evidence for such an array of “benign” Self-Rep information in the Thymus [62].

In principle, V(D)J can randomly imprint in T-cell receptors (TCRs) *any* index arising from recursive function alterations of the *σ*(*g*, *g*) elements with *g* ∈ ***G***. The problem is to ascertain which of the TCR motifs are relevant, novel and potentially dangerous. As noted, large numbers of TCR motifs ranging from 10^15^ to 10^30^ are estimated to be generated for the host in the cortical tissues of the Thymus c-TECs. Of these, only 5% of mature TCRs are actually released from the offline environment of the Thymus into peripheral circulation. “The loss of over 95% of thymocytes reflects the stringent selection processes that shape the developing T-cell repertoire” [59].

In the m-TECs, TCRs are known to undergo first positive selection and then negative selection, having trained, in the latter case, with the self-codes presented to them in the thymic MHC. Therefore, which V(D)J motifs imprinted in the T-cells receptors imply successful selection capable of identifying novel non-self antigens without destroying self-codes? While most models of the AIS selection process for TCRs have relied on a theory of affinity or avidity of the reaction of TCR V(D)J motifs with Self-Rep data in Thymus MHCs (see [76,90]), the G-T-P framework characterizes the issues regarding reactivity as computations. In particular, any software-based change, let alone the hostile biotic malware of the other, is a recursive function transformation from ***Diag***(*g*), *g*  ∈  ***G*** in Self-Rep in Equation (5).

Firstly, the V(D)J operations in the Thymus are simulation exercises conducted in the meta system modelled in Equation (5). In general, as one substitutes different integers for the two-place Gödel substitution function *σ*(*x*, *y*) for given states, the whole space of potential genomic outcomes that can be brought about by recursive functions can be explored in an offline environment, depicted by Table **Ξ**. In Figure 3.

Further, there is an important theorem (see [16,107]) that the *g.ns representing σ(x, y) in the meta-system can always be obtained whether or not the partial recursive function ϕ_x_(y) on the right-hand side of (5) which executes programs halts*. This is essential for the V(D)J and TCR training process to work as simulation exercises.

The diagonal elements *σ*(*g*, *g*) in green in Figure 3 encapsulate the basal information of halting online machine execution of ***Diag***(*g*) = $\phi_{g}\left(g\right)$, viz. of a program *g* that instructs $\phi_{g}$ to run *g* as its input. Therefore, the V(D)J can potentially generate new codes that imply three key changes to $\phi_{g}\left(g\right)$:

(i) A change in the program;
(ii) A change in its input;
(iii) A change in both program and input.

Note that a recursive transformation using a total recursive function *f = ϕ_m_*, *m ∈* ***ℜ*** in Equation (6) for a given *σ*(*g*, *g*) along a row g in Table **Ξ** yields another row with a new index/g.n generically denoted as *f◦g* in order to easily detect the original gene code. By its nature V(D)J motifs where the recursive function *f* is not applied to *g*  ∈  ***G*** but to some *x*∉***G*** can be ruled out a priori as not being biotic. Therefore, large swathes of Table **Ξ** in Figure 3, do not feature in V(D)J. The V(D)J motifs that can be obtained in the cortex of the Thymus can take the following forms for *f = ϕ_m_*, *m* ∈ ***ℜ*** in (6) and *g*  ∈  ***G***:

- *σ*(*g*, *f◦g*): Implying change in input of the basal ***Diag***(*g*) = $\phi_{g}\left(g\right)$.
- *σ*(*f◦g*, *g*): Implying change in program of the basal $\phi_{g}\left(g\right)$ which is no longer a ***Diag***(.) operation.
- *σ*(*f◦g*, *f◦g*): Implying a change in both program and input for ***Diag***(*g*) and transforming it to ***Diag***(*f◦g*).

This set of V(D)J motifs {*σ*(*g*, *f◦g*), *σ*(*f◦g*, *g*), *σ(f◦g*, *f◦g*)} has astronomic number of motifs.

#### 3.3.1. Positive Selection of T Cell Receptor (TCR) Motifs {*σ*(*g*, *f◦g*), *σ*(*f◦g*, *g*), *σ*(*f◦g*, *f◦g*)}

The positive selection process is usually characterized as posing a low bar with the V(D)J motifs only needing “low” affinity to basal gene codes. By this token, while *σ*(*g*, *f◦g*) may appear to have “high” affinity to *g*  ∈ G*. In fact, the motif indicates an online $\phi_{g}$(*f◦g*) operation. However, as *g* is the program for ***Diag***(*g*), hence ***Diag***(*g*) = $\phi_{g}\left(g\right)$ ≠ $\phi_{g}$(*f◦g*). The latter makes V(D)J motifs *σ*(*g*, *f◦g*) untenable in the genomic system and the trivial case where *f◦g* = *g* in *σ*(*g*, *f◦g*) offers no diversity from self. Hence, in this case, all off diagonal terms in rows with *σ(g*, *g)* in the diagonal in Figure 3 are disqualified at the stage of the positive selection of TCRs.

This implies that all V(D)J receptors with motifs {*σ*(*f◦g*, *g*), *σ*(*f◦g*, *f◦g*)} are selected in the process of positive selection. In what follows, the G-T-P bioinformatics will be used to explain how the process of negative selection eliminates all TCRs with V(D)J motifs of *σ*(*f◦g*, *g*) (see [60,108]). The programs in the TCR motif *σ*(*f◦g*, *g*) are the only ones that result in online operations, which are guaranteed to halt and change the basal ***Diag***(*g*) outputs *q* in Equation (4). Hence, if these are released from the mTECs into the periphery they can produce auto-immune disease. Note that the meta-representations *σ*(*f◦g*, *g*) involve off diagonal terms in Table **Ξ** in Figure 3 along row**s** not indexed by *g* but with indexes derived from *g*, viz. *f◦g*.

The next section will give an in-depth analysis of the specific case of *f◦g* programs in *σ*(*f◦g*, *g*) that can involve harmful “negation” transformations to ***Diag***(*g*), which include known non-self antigens *f*^¬^*◦g = g*^¬^ with *g*^¬^ ∈ ***G*^¬^** = $W_{\sigma_{n}\neg }$ in (8) and novel antigens denoted as *f*^¬!^ in (9). These TCR motifs *σ*(*g_n_*^¬^, *g_n_*) will be shown to be most dangerous if released into the periphery. It follows that the fully trained TCRs will have diagonal motifs of *σ*(*f◦g*, *f◦g*) and, in particular, the subset denoted by *σ*(*g_n_*^¬^, *g_n_*^¬^) associated with novel non-self antigen attack, *f_p_*^¬!^ will be shown to be the fixed point given by Gödel sentence for the novel non-self antigen-tissue nexus denoted by {*f_p_*^¬!^, *g_n_*}. The subscript *p* stands in *f_p_*^¬!^ denotes a parasite or hostile other in respect to the host. Thus, successful TCR training will permit the identification of novel antigens that attack genomic outputs *q* online and trigger novel antibody production without harming self-codes.

The significance of why TCR motifs that are released into the periphery take the diagonal *σ*(*f◦g*, *f◦g*) format and, in particular, the *σ*(*g_n_*^¬^, *g_n_*^¬^) formats are three fold. Firstly, as will be shown in the next section *σ*(*f◦g*, *f◦g*), the fixed point that can identify the software-based functions *f = ϕ_m_*, *m* ∈ ***ℜ*** and the basal ***Diag***(*g*) program online will be transformed to ***Diag***(*f◦g*). Secondly, as discussed in [45], in the context of a game with self (host) and the other (pathogen), only diagonal elements demonstrate Nash equilibria when both status of self and self’s identification of non-self status are in sync, with false beliefs and undetected deceit being ruled out. As indicated in the case of the Interferon Gamma knock out in Figure 1, when the first place marker on the left in *σ*(*g_n_*^¬^, *g_n_*) has been updated, but the second place marker for the host’s beliefs on the agency of the other cannot be synced, this is a cause for immune failures. Technically, without both place markers in sync, as in *σ*(*g_n_*^¬^, *g_n_*^¬^) in the case for novel antigen attack *f_p_*^¬!^, the Gödel sentence cannot be successfully generated to identify *f_p_*^¬!^ and hence the genomic system cannot respond with the novel antibodies.

#### 3.3.2. Synchrony in Anticipatory T-Cell Receptors Clone of Tissue Specific Attacker with Peripheral MHC Record of Same in Online Attack

The objective of this section is to show how successful TCR motifs simulated and selected in the offline m-TECs on basal Self-Rep data for the host of malware $f_{p}^{\neg }$ attack on specific gene codes *g_n_* have to synchronize with identical motifs in the *peripheral* MHC receptors, which are recorded, should the same *f_p_*^¬^ antigen attack online and in real time. The G-T-P logic is necessary to show that this synchrony involves a fixed point for which the Rogers Fixed Point Theorem [16] (Section 11.2) and also Second Recursion Theorem [17] (p. 200) will be used.

#### 3.3.3. Negative Selection of T-Cells

The offline training of the T-cells is ultimately for detection and elimination, in the online environment that immunologists call peripheral tissues, of malware (non self-antigen) that attacks gene codes in cells of tissues. The T-cells have to counter malware without attacking self-codes, which cause autoimmune disease. There have been extensive discussions about the elimination of self-reactive T-cells in m-TECs to avoid autoimmune disease. However, as noted by [90] (see [109]), with little or no focus on how T-cell training equips T-cells for “self non-self discrimination that continues in the periphery after thymic negative selection, this is an enigma.”

The point that is not sufficiently understood in these discussions that can be made clear in the G-T-P logic are the two requirements: (i) the proper training for T-cells so that they do not carry clones of lethal non-self antigens that can then attack self-codes and, (ii) the *peripheral* MHC antigen receptors record the online attack in real time, such that the encoded information in the T-cell receptor and the peripheral MHC satisfy the composite encoding for a unique fixed point with regard to the novel malware *f_p_*^¬!^ attacking a tissue specific gene code *g_n_*. This encoding, as has been indicated, is the famous Gödel sentence and its formation for the novel antigen-tissue nexus denoted by {*f_p_*^¬!^, *g_n_*} will be vital for the genomic system to produce novel antibodies to counter the pathology from {*f_p_*^¬!^, *g_n_*} with precision.

To explain the G-T-P bio-informatics behind this, I will present the case when a non-self antigen attack $f_{p}^{\neg }$ on *g_n_* succeeds. The following so called Liar Strategy equation, first shown in Markose [45], is needed for this:

(10) $$\phi_{f_{p}\neg !Diag\left(g_{n}\right)}\left(s\right)=\phi_{\sigma ({g_{n}}^{\neg },g_{n})}=\phi_{\phi_{g_{n}^{\neg }}\left(g_{n}\right)}\left(s\right)=\neg \phi_{\phi_{g_{n}}\left(g_{n}\right)}\left(s\right)\;=q\neg \;iff\;\phi_{\phi_{g_{n}}\left(g_{n}\right)}\left(s\right)=q.$$

#### 3.3.4. Dangerous V(D)J Codes and Successful Non-Self Antigen Attacks

The first term on the LHS of (10) has the novel antigen *f_p_*^¬!^ attack the online basal halting ***Diag***(*g_n_*) *=* $\phi_{g_{n}^{\;}}\left(g_{n}\right)$ and it succeeds in negating this if and only if (*iff*) the halting computation is in place (viz. the *q* tissue or the regulatory factor are in situ as in the first term on the RHS of (10)) for *f_p_*^¬!^ to attack online. Note that, also in the first term on the left in (10), the offline index in the periphery MHC antigen receptor, relating to real time pathogen activities, is in default mode with the P-MHC meta index *σ*(*g_n,_*, *g_n_*), mirroring the ***Diag***(*g_n_*) benign state of health.

Now we turn to the motif generated offline in the Thymic TCR. Following the index function convention for *σ*( *,* ) in (5), the index *g_n_*^¬^ derived in the V(D)J represents the change brought about by *f_p_*^¬!^***Diag***(*g_n_*) for which the TCR motif is *σ*(*g_n_*^¬^, *g_n_*). Thus, the latter TCR motif is the index for the simulation of $\phi_{\phi_{g_{n}^{\neg }}\left(g_{n}\right)}\left(s\right)$ in (10) and, therefore, represents the case when the pathogen *f_p_*^¬!^ has “highjacked” the program *g_n_* online and, having altered it to *g_n_*^¬^, uses the original *g_n_* as an input to produce outcome *q*^¬^, which is antithetical to the original q-based tissue.

Finally, in (10), the TCR motif for $\phi_{\phi_{g_{n}^{\neg }}\left(g_{n}\right)}\left(s\right)\;$, the two place meta/mirror index *σ*(*g_n_*^¬^*, g_n_*) could be interpreted as follows—the first place *g_n_*^¬^ states that the {*f_p_*^¬!^, *g_n_*} attack could take place, but the second place index in the meta system of the host does not assign malign agency to the other. If this TCR motif escapes negative selection and is released into the periphery, it will identify the self-assembly ***Diag***(*g_n_*) and then inflict the damage entailed in *f_p_*^¬!^ as in *¬*$\phi_{\phi_{g_{n}^{}}\left(g_{n}\right)}\left(s\right)\;$*= q*^¬^. This is the same as if an online attack with $\phi_{\phi_{g_{n}^{\neg }}\left(g_{n}\right)}\left(s\right)$ in (10) has taken place. In other words, the insufficiently trained T-cells with the *off-diagonal* motif *σ*(*g_n_*^¬^, *g_n_*), as shown in Figure 3 in row *g_n_*^¬^ of Table **Ξ** will be just as lethal as if the non-self antigen of the same ilk had attacked online.

As noted in [45] with regard to the Liar strategy, here, the malware/pathogen also succeeds only out of equilibrium in (10) with the malware *f_p_*^¬!^, altering the gene code *g_n_* code to *g_n_*^¬^ under conditions when the host has not yet updated the second place *g_n_* in *σ*(*g_n_*^¬^, *g_n_*) to reflect the self-identification of the agency of the hostile other. On the flip side, from the perspective of pathogen, the success of *f_p_*^¬!^ requires that the host is deceived or, in terms of the Interferon Gamma knock-out discussion, the host has a blind spot regarding the other. Indeed, it is well known that viruses try to deactivate the Interferon Gamma in the peripheral MHC in order to evade detection by the AIS [76].

#### 3.3.5. Negative Selection of Dangerous T-Cells Receptors with Motifs: *σ*(*g_n_*^¬^, *g_n_*) in (10)

Thus, the situation in (10) is dangerous in two respects. T-cell Receptors with motifs such as *σ*(*g_n_*^¬^, *g_n_*) in (10) are dangerous if they escape negative selection and are released into the periphery as they will accomplish the negation of the tissue specific gene code *g_n_*, as shown in (10), viz. bring about autoimmune disease. It is conjectured that TCR motifs of *σ*(*g_n_*^¬^, *g_n_*) are eliminated at the stage of negative selection due to their capacity for generating autoimmune pathologies, as can be formally confirmed in Equation (11), that *f_p_*^¬!^ will result in ¬$\phi_{\phi_{g_{n}}\left(g_{n}\right)}\left(s\right)$, the negation of basal self-assembly outputs:

(11) $$\phi_{\sigma \;\left(g_{n}^{\neg }{,\;g}_{n}\right)\;}\left(s\right)=\;\phi_{\phi_{g_{n}^{\neg }}\left(g_{n}\right)}\left(s\right)\;=\;\phi_{f_{p}\neg !Diag\left(g_{n}\right)}\left(s\right)=\;\neg \;\phi_{\phi_{g_{n}}\left(g_{n}\right)}\left(s\right)\;\;\mathrm{as}\;Diag(g_{n})\;=\;\phi_{g_{n}^{\;}}\left(g_{n}^{\;}\right).$$

The only TCR motifs that survive the Thymic training are the updated versions of *f_p_*^¬!^ ***Diag***(*g_n_*^¬^) commensurate to the online function $\phi_{g_{n}^{\neg \;}}\left(g_{n}^{\neg }\right)$ for which the V(D)J/Self-Rep index yields *σ*(*g_n_*^¬^, *g_n_*^¬^).

Secondly, this should match the encoding in the peripheral MHC antigen receptors when there is an actual attack of the tissue specific gene codes. For this to happen post attack by *f_p_*^¬!^ on ***Diag***(*g_n_*), the P-MHC has to update the offline real time receptor to ***Diag***(*g_n_*^¬^) *= σ*(*g_n_*^¬^, *g_n_*^¬^). In other words, the host’s immuno-cognitive system must encode the Gödel sentence in Equation (12). For this, the variant of the Second Recursion Theorem, called Rogers Fixed Point Theorem ([16] Section 11.2 and [17]), is used (see Endnote [110] for proof). Note that the index function *σ*(*g_n_*, *g_n_*) in the Self-Rep operation for ***Diag****(g_n_*) = $\phi_{g_{n}^{\;}}\left(g_{n}\right)$ from Equation (5) satisfies the first step in the derivation of the fixed point for a total computable function. The index *σ*(*g_n_*^¬^, *g_n_*^¬^) represents the function $\phi_{g_{n}^{\neg \;}}\left(g_{n}^{\neg }\right)$ that is obtained by substituting the index *g_n_*^¬^ for *f_p_*^¬!^ ***Diag***(*g_n_*) in ***Diag***(*g_n_*). Set *v* to be the g.n of ***Diag***(*g_n_*^¬^) *=* σ(*g_n_*^¬^, *g_n_*^¬^) *=* $\phi_{g_{n}^{\neg \;}}\left(g_{n}^{\neg }\right)\;$(see [110]), by construction, *v* is the fixed point of the malware/Liar function *f_p_*^¬!^. This is shown in the diagonal array of Figure 3 of the *g_n_*^¬^ row of Table **Ξ**. This yields:

*The Gödel Sentence for T-Cell Receptor and Peripheral MHC fixed point for {f_p_^¬!^, g_n_*}

(12) $$\phi_{f_{p}^{\neg }!\left(v\right)}(s)≅\;\phi_{f_{p}^{\neg }!\sigma \left(g_{n}^{\neg },g_{n}^{\neg }\right)}\left(s\right)\;\;≅\;\phi_{f_{p}\neg !Diag\left(g_{n}^{\neg }\right)}\left(s\right)≅\phi_{\phi_{g_{n}^{\neg }}\left(g_{n}^{\neg }\right)}(s)≅\phi_{\sigma \left(g_{n}^{\neg },g_{n}^{\neg }\right)}\left(s\right)≅\phi_{v}(s).$$

Thus, we have the synchrony of the T-cell receptor motif of the fixed point *σ*(*g_n_*^¬^, *g_n_*^¬^) of malware function *f_p_*^¬!^on the R.H.S. of (12) with the peripheral MHC antigen receptor update for the same on the L.H.S. of (12). The index *σ*(*g_n_*^¬^, *g_n_*^¬^) is a very precise self-referential statement regarding which gene code is under attack and the biotic identity of the pathogen that is attacking it. There is now a remarkable transformation of the message in *σ*(*g_n_*^¬^, *g_n_*) of (10), which effectively gives the TCR instructions to attack self-code *g_n_*, to *σ*(*g_n_*^¬^, *g_n_*^¬^) in the fixed point of the Gödel sentence which says *g_n_* is under attack by the hostile other. Further, by construction, the fixed point *v* = *σ*(*g_n_*^¬^, *g_n_*^¬^) of *f_p_*^¬!^ implies that in $\;\phi_{f_{p}^{\neg }!\left(v\right)}≅$ $\phi_{v}$(*s*) both sides are undefined and represent non-halting computation as assuming otherwise will produce a contradiction (see Endnote [111]). Hence, in Equation (12), the output of the game is not predictable. At this juncture, whether the pathogen or host will win is undecidable, once the host has identified the hostile agency of the other. This implies, from Post [37], that the productive construction of the set ***G*^¬^** in (8) and Figure 2 follows in that the index *τ*(*g_n_*^¬^) *= σ_n_*^¬^ for the Gödel sentence will lie outside the two listable or recursively enumerable disjoint sets, respectively, for the “theorems” of the system, ***G***, and the known list of “non-theorems”, ***G*^¬^** = $W_{\sigma_{n}\neg }$.

There is a very important point about how it is that the Gödel sentence works to successfully trigger a novel antibody response to a counter the new *f_p_*^¬!^ non-self antigen. The theoretical anticipatory leg of the fixed point generated in the Thymus trained T-cell receptors from basal information on the far right of (12) must align with the online experientially driven component of the fixed point in the peripheral MHC receptors index in the first term on LHS generated from $\phi_{f_{p}\neg !Diag\left(g_{n}^{\neg }\right)}\left(s\right)$ when the {*f_p_*^¬!^, *g_n_*} attack happens.

**Figure 4:**
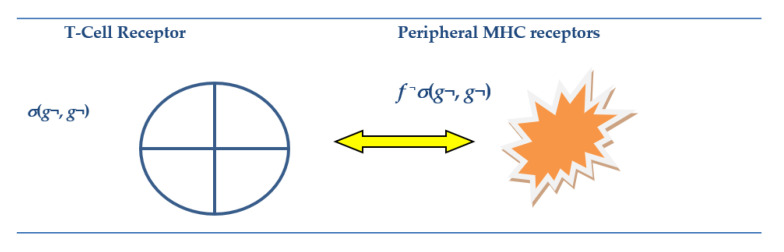
Gödel Sentence In Action: The T-Cell Receptor (TCR) and the Peripheral MHC (P-MHC) fixed point for novel non-self antigen- tissue gene code pair {*f_p_*^¬!^, *g_n_*}; experiential record of attack in peripheral MHC (RHS) syncs with theoretical/anticipatory T-cell receptor clone for *f_p_*^¬!^ (LHS) viz. $\phi_{f_{p}\neg !\sigma \left(g_{n}^{\neg },g_{n}^{\neg }\right)}\left(s\right)≅\;\phi_{\sigma \left(g_{n}^{\neg },g_{n}^{\neg }\right)}\left(s\right)$ in Equation (12).

If the online attack does not take place, the successfully trained T-cell receptors with motifs *σ*(*g_n_*^¬^, *g_n_*^¬^) will see no action. The information in the T-Cell generation of the fixed point for the *f_p_*^¬!^ was done speculatively by the V(D)J. The successful positive and negative selection then permits the T-cells to go into peripheral circulation in the vicinity of the tissue in question with the mug-shot (code) of the attacker effectively in its cross hairs, is shown on the left hand side of Figure 4.

Figure 4 shows how the successful rendezvous for the two legs of the fixed point for the non-self antigen *f_p_*^¬!^ occurs; that is, the anticipatory Thymus trained T-cell receptor for the self-other pair {*f_p_*^¬!^, *g_n_*} on the left of Figure 4 and the experiential record, when the real time attack by *f_p_*^¬!^ happens, in the peripheral MHC receptor on the right. Note, the update of the P-MHC receptor meta index from the default mode of basal health of *σ*(*g_n_*, *g_n_*) to the meta index *σ*(*g_n_*^¬^, *g_n_*^¬^) after the attack in order for the digital code of the hostile other is recorded is known to be governed by the Type 1 Interferon Gamma (see [76,91,93]). 

### 3.4. Precision Engineered Novel Antibodies Made Possible Only by Gödel Sentence σ(g_n_^¬^, g_n_^¬^)

In summary, the fixed point in Equation (12) permits the specific gene code of the host to self-report that it is under attack by a novel non-self antigen (the hostile other), and this plunges the genomic system into a state of radical uncertainty in the form of undecidability. For the Gödel sentence in (12) to form in the organism, the record of the attack that has taken place must be updated in the P-MHC and syncs with the T-cell speculation that it could take place in the precisely stated way in (12). At this point, the adaptive immune system of the host is geared toward countering the malware. For this, a new antibody has to be produced and then applied en-masse (see [112] and [113]). The host is compelled by G-T-P logic of the Gödel sentence *σ*(*g_n_*^¬^, *g_n_*^¬^) to adopt the *only* best response function logically permitted by the G-T-P framework. This is the Post [37] productive recursive surprise strategy function [45], of the host (*h*), *f_h_*^!^ in that it will have to be an innovation, viz outside of known extant machine listable sets in Figure 2:

(13) $${f_{h}}^{!}(\sigma ({g_{n}}^{\neg },\;{g_{n}}^{\neg }))\;\mathrm{with}\;{f_{h}}^{!}\;=\phi_{m},\;\mathrm{such}\;\mathrm{that}\;m\;\in \;ℜ\;-\;(G\cup \;G^{\neg })$$

The innovative antibody is precision engineered by the B-cells from the information in *σ(g_n_*^¬^, *g_n_*^¬^). This will be contrasted with generic responses to the record of a pathogen based on the innate immune system.

In Markose [45] (Lemmas 3 and 5), it is shown how a non-trivial Post [37] recursive reduction function from the indexation of the undecidable proposition from the Gödel sentence in (12) given as *τ*(*g_n_*^¬^) = *σ_n_*^¬^ in Figure 2, will result in novelty production in a Nash equilibrium, viz. a recursive surprise function for the host, *f_h_*^!^. In the case of the adaptive immune system, this takes the form of new antibodies. Corresponding to the set $W_{\sigma_{n}\neg }$ in Figure 2, the Post [37] recursive reduction that implements the surprise strategy function will be indexed as *σ_n_*^!^ such that the surprise/novelty strategy set $W_{\sigma_{n}!}$ satisfies the consistency requirements of the basal information in Figure 2, viz. *σ_n_*^!^ can only be added to $W_{\sigma_{n}!}$ and cannot belong to $W_{\sigma_{n}!}$. This is shown to have the structure of a co-evolutionary arms race with innovative antibodies that can ensue as a Nash equilibrium in which both host and pathogen coexist.

It can be conjectured that the Post [37] theory of indexes of sets of the domain (or range) of recursive functions (see also [38]) where the powerful idea of recursive reduction operations outlined above and in [44] in the context of genomic intelligence, govern all RNA regulatory networks so that relays of digital on–off switches satisfy the original basal organization of listable genomic “theorems” in set ***G*** and the non-theorems there-off in set ***G***^¬^ = $W_{\sigma_{n}\neg }$ in Figure 2.

The arms race in the immune system is exactly that—the immune system is primarily evolving its defensive tactics against biotic hackers, which aim to “highjack” gene codes of the tissue specific cells to do their bidding. Genomic identity and somatic integrity, which have continued over the millennia, is the remarkable consequence of the immuno-cognitive system being able to put in place a gene code centric cyber security. The spectacular horizon scanning done by the adaptive immune system and the decentralized nature of biotic cyber defense are other notable features of the system.

### 3.5. G-T-P Bioinformatics for COVID-19 Pathology and Recovery

It can be conjectured that the major reason for the breakdown in the adaptive immune response in the generation of novel antibodies precision engineered for the novel pathogen-tissue attack, denoted by {*f_p_*^¬!^, *g_n_*}, is as follows. In the case of COVID-19 pathogen, the tissues *g_n_* relate to respiratory organs. As indicated, a novel non-self antigen will have to attack online for the experientially driven peripheral MHC receptor to update the meta index from the default mode of basal health of *σ*(*g_n_*, *g_n_*) to the meta index *σ*(*g_n_*^¬^, *g_n_*^¬^) needed for the fixed point setting of $f_{p}^{\neg }!\sigma \left(g_{n}^{\neg },g_{n}^{\neg }\right)$ for the novel antigen in the first term on the left-hand side of (12). Despite, its prodigious outputs, as V(D)J in T-cell receptors have inbuilt stochasticity, it is possible that the T-cell cloning did not produce the code *g_n_*^¬^ for {*f_p_*^¬!^, *g_n_*} novel pathogen-tissue pair. However, there is growing evidence that it is a deficiency in the Interferon Gamma circuitry [114]—to assign, as per the G-T-P conjecture, the update in the P-MHC meta index to *f_p_*^¬!^*σ*(*g_n_*^¬^, *g_n_*^¬^) to record the online post attack state of $\phi_{f_{p}\neg !Diag\left(g_{n}^{\neg }\right)}\left(s\right)$. This implies that the immune system, instead of going toward novel targeted antibody production from the formation of the Gödel sentence in (12) will instead produce generic innate immune response of a plethora of analog defenses, such as inflammation, toxicity, ingestion of pathogens, etc. The latter is called a “cytokine storm”. A run-away manifestation of this cytokine storm and a marked absence of COVID-19 antibodies from novelty producing somatic hypermutation has been found to be the hallmark of the pathology in many who have succumbed to COVID-19 [91,93].

Studies have found that there is a deficiency in Type 1 Interferon Gamma in many who have suffered COVID-19 morbidity from either congenital defects [91], or from self-triggers of antibodies that destroy Type 1 Interferon Gamma [115]. G-T-P logic indicates that Type 1 Interferon Gamma deficiency is a top contender for what is preventing the peripheral MHC receptor from achieving the updates to the meta record of the hostile other for the full generation of the Gödel sentence which is a logical necessity for production of novel software-based antibodies for COVID-19 pathogen-tissue pair.

## 4. Conclusions

Notwithstanding the “riddle of the code” [116], which relates to a lack of consensus on the prebiotic conditions that led to the four-letter nucleic acid-based encoding of genetic material, the digitization of inheritable information in the genome has set in motion what [117] have called the “algorithmic takeover” in biology. However, few have sought to fully spell out the necessary refinements for software-based genomic systems that correspond with the epochal foundational work of Gödel-Turing-Post (G-T-P).

As discussed, and subsequently summarized in Table 1, many studies [64,65,66,67,68,70,71] underscore the intricate recognition of the self and the other, especially the hostile other, in the workings of the adaptive immune system and adduce the same molecular and neuro-physiological underpinnings for social cognition and complex strategic behaviors. Indeed, there has been a liberal use of metaphors for “mirrors” and also of self-reference to describe findings on structures in the adaptive immune system (see, [59,60,62,70], to name a few) and also in the Mirror Neuron System (MNS) in the brain discovered by the Parma group [47,48,49]. However, perhaps apart from [44,45,46], few have explicitly made the link between the Recursive Function Theory operations of Self-Ref and Self-Rep and the mirror/meta systems found in the adaptive immune system and the Mirror Neuron System. A prominent G-T-P operator, ***Diag***(*x*) where a program *x* instructs the machine execution of itself, *x*, has been shown to characterize gene codes as self-assembly programs [85] and can be conjectured to be biotic software that is generic to genomic intelligence. This paper has used the Rogers Meta-Representation Theorem from [16] in Equation (5) to formalize the key phenomena of online machine executions involving ***Diag***(.) and the offline record of the same as being a significant evolutionary development in genomic information processing of eukaryotes that correlates with their complexity. Figure 1 is useful to show how there is evidence for an identical G-T-P mirror recursive machinery in the immuno-cognitive systems to make a 1–1 mapping between online and offline activities and also to manage the nexus between self and the other by reusing self-codes to identity the other. The finding of Major Histocompatibility Complex (MHC) related gene expression in the healthy brain was initially deemed “unexpected” [73], due to the association with Thymic MHC for T-cell training and non-self antigen detection in the adaptive immune system. It is being posited that MHC gene expression in the brain (see [73,74]) is not there to facilitate an immune response to pathogens, but because identical software is used for the self-other nexus in both AIS and social cognition in MNS (see Table 1 (3a, 3b). Hence, as indicated in Figure 1 the knockout of the Interferon Gamma regulator circuitry for the other in the MHC related self-other nexus, gives a causal reason for the impairment of social cognition as well as of the immune system.

The capstone of the recursive structures in G-T-P computation is a syntactic construction called the Gödel sentence arising from a fixed point involving variants of a malign viral software [44,45]. To date, the syntactic encoding of the Gödel sentence is the only known way for endogenous novelty production in digital systems [44,45,54]. Novelty comes from the fact that the Gödel sentence lies outside two disjoint machine listable set of theorems and non-theorems for that system, as seen in the Post [37] set theoretic proofs of Gödel incompleteness illustrated in textbooks such as [17] and adapted in Figure 2. The logician’s depiction of the Gödel sentence as a code based entity in a formal system that asserts that it is neither provable nor refutable (see [77,118]) belies the unique construction of the Gödel sentence and its ubiquity in genomic systems of eukaryotes. In the context of a genomic game between gene codes and the malign viral software, the Gödel sentence gives a step-by-step construction of how a code-based system with the necessary G-T-P machinery will permit a code to self-report that it is under attack by a non-self antigen. The explicit assertion of the malign agency of the other which is tantamount to identifying the fixed point of the novel viral software, has been shown to be paramount in the construction of Gödel sentence and for the adaptive immune system to produce novel antibodies. As indicated here, but developed further in Markose [45], novelty production requires an operation of a computable function based on the Gödel sentence, which Post [37] calls a productive function, as it will map *outside* extant listable sets of genomic indexes of known algorithms for that system.

The adaptive immune system which embarks on a prodigious number of V(D)J recombinations in the T-cell receptors trained on mirrored Self-Rep expressions of their own gene codes then self-referentially produce digital signatures of clones of potential hacker malware in an offline meta environment of the Thymus. The Thymic offline Self-Representation of online halting machines is shown to be a textbook case of G-T-P recursive meta/mirror systems. Most models of the AIS selection process for TCRs have relied on a theory of affinity or avidity of the reaction of TCR V(D)J motifs with Self-Rep data in Thymus MHCs (see [76,90]). The G-T-P framework characterizes the issues regarding reactivity and successful TCR selection as involving computation, with precise V(D)J codes given in Equation (12) for the Gödel sentence for novel non-self antigen. Despite, explicit recognition that bio-molecular computation with information being encoded in molecules leads to issues of cybersecurity [30], this is the first time a precise code-based signature has been derived for the TCR to identify a novel non-self antigen. The remarkable coming together in the construction of the Gödel sentence, see Equation (12) in Section 3.4, of the theoretical and anticipatory cloning of putative malware in the T-cell receptors with the experientially driven record of the same in the peripheral MHC receptor if and when the novel pathogen attacks in real time, clearly dispenses with any anti-computationalism [78] from oracles or deus ex machina. While deficiencies in AIRE factor for mirroring self-gene codes can impair adaptive immunity, the G-T-P analyses of COVID-19 pathology in Section 3.5 shows how the Type 1 Interferon Gamma deficiency can create a blind spot in the self-other nexus in the peripheral MHC. This has a critical bearing on the loss of capacity in an individual to respond with novel antibodies from somatic hypermutation to counter COVID-19 infection. Studies [91,93,115] have found that either a congenital deficiency or an auto-immune knockout of Type 1 Interferon Gamma creates a blind spot on the agency of the other in the peripheral MHC detection of the attack. As per the G-T-P logic, this affects the construction of the Gödel sentence and hence results in a cytokine storm of innate immune system response rather than the production of novel antibodies for COVID-19.

No doubt, the genomic intelligence of the AIS is wedded to open-ended search and primed for the dark arts of non-self antigen creation that could lead to auto-immune disease. This has led some to wonder if “such an anticipatory system of defense is more trouble than it is worth” [87]. What has been underscored in this paper is that, in the absence of the 3 G-T-P conditions for Gödel incompleteness being hardwired in the eukaryote immuno-cognitive system, “thinking outside the box”, strategic innovation involving self-other interaction and an arms race in novelty production are not possible. In the context of extended phenotypes, to use a term coined by Dawkins [119] to refer to artefacts developed external to the organism, the evidence for a cognitive G-T-P mirror based recursive machinery in all eukaryotes, which reaches its apogee in humans, supremely prime them for empathic, Machiavellian and highly protean extended phenotypical behaviors [50,120].

It has been pointed out in Markose [44,45,104,121] that such G-T-P games are outside the purview of standard game theory as was first critiqued by Binmore [122] in that the action set is closed and complete and novelty and surprises (in phenotypes) are considered not to be a Nash equilibrium of a game [123,124]. In contrast, the starting point of a genomic AIS G-T-P game is to identify novel malware for which the search begins *outside* of extant machine listable sets of encoded information of known phenotypes/algorithms. The Nash equilibrium of the AIS G-T-P game has to first construct the Gödel sentence for the host to produce novel antibodies that entails a coevolutionary arms race of novelty production by host and pathogen that is fully characterized by the Post [37] productive recursive functions as first noted in [45].

The leading model of genomic novelty is based on transcription errors. This has made it difficult for complexity and decision sciences to integrate (see [44]) the far-reaching implications of the major paradigm shift that has followed the Nobel prize winning work of Barbara McClintock (championed by [13,24,25,26,27,28,125], amongst others) on the role of viral software in the form of transposable elements in the production of genomic variety and in the evolution of complexity. One aspect of genomic complexity is in the circuitry of the RNA regulatory networks which are mapped in the different segments of the genome by transposable elements manifesting a high incidence of repeat sequences. This can be conjectured to be the consequence of the organization of gene regulatory networks as a distributed ledger where each transcription binding site contains codes of all others in the regulatory network in consistent off or on positions dictated by the recursive reductions relating to the creative and productive sets in Figure 2 of genomic basal codes. Recursive reductions are algorithmic functions formulated by Post [37] that keep systemic consistency between “derived” recursively enumerable sets and the basal archetypal sets of genomic codes as theorems and non-theorems of the system responsible for the somatic integrity of the organism. The distributed ledger technology in implementing synchronous updates at all binding sites or nodes of a given gene regulatory network vitiates asymmetric information and scope for maladaptive signaling games [126] *within* the organism. As indicated, a unifying computational framework that befits a post McClintock era of biology is only starting.
